# A Monte Carlo simulation study comparing the up and down, biased-coin up and down and continual reassessment methods used to estimate an effective dose (ED_95_ or ED_90_) in anaesthesiology research

**DOI:** 10.1016/j.bjao.2023.100225

**Published:** 2023-09-27

**Authors:** Jean-François Fils, Panayota Kapessidou, Philippe Van der Linden, Emmanuel Guntz

**Affiliations:** 1 Ars Statistica, Nivelles, Belgium; 2 Department of Anesthesiology, University Hospital Saint-Pierre, Université Libre de Bruxelles (ULB), Brussels, Belgium; 3 Faculty of Medicine, Campus Erasme, Free University of Brussels, Brussels, Belgium; 4 Department of Anesthesiology, Hôpital Braine-l’Alleud Waterloo, Université Libre de Bruxelles (ULB), Braine-l’Alleud, Belgium

**Keywords:** biased-coin up and down, continual reassessment method, dose-finding, effective dose, up and down, Monte Carlo simulations

## Abstract

**Background:**

Dose-finding studies in anaesthesiology aim to target the effective dose (ED) of an anaesthetic agent in a specific population. The common dose-finding designs used are the up and down method (UDM), the biased-coin up and down (BCD), and the continual reassessment method (CRM). Although the advantages of CRM over the UDM and BCD methods have been described in the statistical literature in terms of precision and direct estimation of ED, CRM may also offer attractive properties from an ethical point of view.

**Methods:**

Based on Monte Carlo simulations, this article aims to compare the three methods with regard to 1) their ability to find as close an estimate as possible for the ED_95_ or ED_90_ and 2) the total number of patients needed to treat and the number of failures.

**Results:**

In contrast to BCD and UDM, CRM does find an estimate for ED_95_ and ED_90_. UDM underestimates both ED_95_ and ED_90_. BCD is close to the targeted EDs when the starting dose does not exceed the ED of interest, otherwise it overestimates it. CRM with cohorts of two patients is closest to the ED of interest independently of the starting doses. CRM requires between 20 and 50 observations, UDM should include 90 patients, and BCD 100 or 60 observations. Lastly, CRM is associated with fewer failures, compared with BCD and UDM.

**Conclusions:**

Based on Monte Carlo simulations, our work suggests that the UDM is not an adequate dose-finding method because it underestimates the ED of interest. Compared with BCD, CRM offers the advantages of being more efficient, requires fewer patients to be included, and is associated with fewer failures.

Dose-finding methodology was mainly developed for drug candidates in oncology trials, where it is of crucial importance to define the dose that is both effective (i.e. improving or curing the intended disease condition) and safe (with an acceptable risk of adverse events).^1^ In oncology, the goal of a dose finding phase I trial is to target the minimum effective dose (MED) (i.e. doses in the range of 20–33% of effectiveness).^2^

In anaesthesiology, the currently applied dose-finding designs aim to target doses in the range of 90% (ED_90_) to 95% (ED_95_) of effectiveness. Three designs have been described and adopted by anaesthesia researchers: the up and down sequential allocation method^3^^,^^4^ (UDM), the biased-coin up and down (BCD),^5^ and the continual reassessment method^6^ (CRM). CRM represents an adaptive Bayesian design that uses all available data before the trial's onset and data collected during the trial. Therefore, the model ‘learns’ from data gathered throughout the study, with all recruited patients contributing to the estimated dose/volume/concentration. As a result, CRM offers a direct and unbiased^7^^,^^8^ estimate of the ED_95_ and ED_90_ and is therefore better suited to target the ED_95_ than the two other models, as reported in the statistical literature.^9^, ^10^, ^11^

CRM could also offer more advantages regarding the number of patients to be included in the studies and the number of observed failures, which is often poorly reported with the other models. Despite its statistical advantages, CRM remains less frequently used in anaesthesiology, compared with the UDM or BCD designs. This might be related to the fact that conducting a trial with a CRM design requires more professional statistical expertise than for the UDM or BCD models.

The primary objective of this article was to compare the three designs used to determine a targeted ED, on two key elements:

1) the ability and the accuracy of the method to estimate an ED_95_
2) the total number of patients needed AND the number of failures.

The secondary objective was to compare the three designs on the same key elements, but with a targeted ED_90_. Both ED_95_ and ED_90_ are the EDs most appropriate to target in anaesthesia research.

To achieve these goals, Monte Carlo simulations have been drawn for each design and condition and results have been compared on the criteria described above. A review of the three dose-finding designs is presented in Appendix 2.

## Methods

### Rationale for a simulation study and 25 doses tested explored

The three designs (UDM, BCD, and CRM) have been tested on predefined dose-response relationships, determined in advance. The goal of this study was to test the ability of the three designs to detect the ED of interest (ED_95_ and ED_90_) when the true EDs are known and when the range of possible doses comes near to a real possible phase I clinical trial in anaesthesiology.

When comparing CRM to other designs or studying the behaviour of CRM, authors generally used a limited set of doses (usually no more than six^7^^,^^12^^,^^13^), although, in real phase I dose-finding designs, up to 15 doses can be explored.^14^^,^^15^ Therefore, we decided to study dose-response curves with 25 possible doses, reflecting a higher number of possible doses for a dose-finding study, equally spaced in volume; that is, an equal volume difference is assumed between all doses (e.g. 0.5 μg of an anaesthetic agent).

Monte Carlo simulation is a widely used mathematical tool to simulate and study the behaviour of mathematical algorithms under various conditions. In this study, we generated 5000 estimations of ED_95_ and ED_90_ for each design and tested whether the designs were able to target the ED of interest. We also reported the number of observations and failures observed. All doses have been used as the possible starting dose of a dose-finding trial, reflecting situations where the clinician may or may not have knowledge of the dose-response relationship. This also allows a complete comparison of the designs across the tested doses, even if, for instance, it is recommended for the CRM to start the trial in the ‘expected’ range of the ED of interest. R software (R Foundation for Statistical Computing, Vienna, Austria) and its packages cir, plyr, drc, dplyr, and car were used to determine the dose-response curves and to perform the 5000 simulations drawn by each design, using a predefined seed, allowing reproducible results. Appendix 1 includes the R code to reproduce the Monte Carlo results, detailed in the subsections of this article dealing with UDM, BCD, and CRM.

### Dose-response functions definition

**Fig 1:**
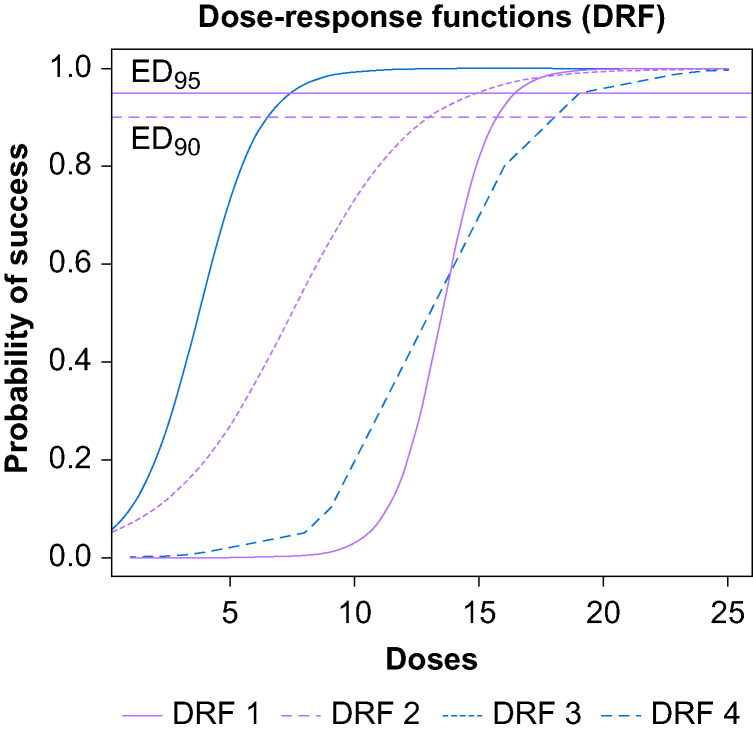
Dose-response functions definition.

Three dose-response functions (i.e. dose-response relationships) comprising 25 possible doses, equally spaced in volume, were drawn on the interval (0–100%) to obtain a theoretical dose associated with the ED_90_ and the ED_95_. A probability of success (successful response) is associated with each dose for the three dose-response functions, allowing the ED_50_, ED_90_, and ED_95_ to be known *a priori*. The first dose-response curve was generated through an exponential equation (y=1/(1+exp [−x]), and the second and the third ones were built using a four-parameter log-logistic model (c/(1+a∗exp(-b∗t)), where a=20, b=0.1, c 00, d=0 for the second one and where a=20, b=0.075, c=100, d=0 for the third one, where t is a vector of numbers comprised between 1 and 100, with an incremental step of 0.0001 between the numbers). These three dose-response functions were used to compare the three designs. A fourth dose-response function was built manually by the first author and used to calibrate the random walk of the CRM (see Appendix 2). Based on these three dose-response functions, the ED_95_ and ED_90_ were obtained for function 1 (16.44445 and 15.69725, respectively), function 2 (14.85042875 and 12.98239125, respectively), and function 3 (19.8005713 and 17.3098563, respectively). The success probabilities by dose and by dose-response function are depicted in Fig 1 and Table 1 (see Appendix 2).

**Table 1:** How to use the continual reassessment method in anaesthesiology? CRM, continual reassessment method; ED, effective dose.

Start the CRM around the ED of interest (ED_95_ or ED_90_)	Always start the CRM with the dose thought to be associated with the targeted ED (e.g. ED_95_ or ED_90_). The working model will comprise doses around this value (higher and lower doses).
**Random walk between two CRMs**	If the CRM fails to find an estimate for ED_95_ or ED_90_ a) no failure observed for the first CRM, suggesting a start at a too high dose; b) >25% of failures; c) *a posteriori* probabilities for all doses too high (>0.925 for ED_95_ and 0.875 for ED_90_) or too low (<0.75 or 0.70 for ED_95_ and ED_90_, respectively), stop the current CRM and restart another dose-finding study using a higher or lower set of doses, as suggested by our random walk definition based on the observed success probabilities of the current CRM (see Appendix 1).
**Cohort size**	Two-patient cohorts give better estimations of the ED_95_ and ED_90_ than cohorts of four patients.
**Dose selection for the next cohort of patients**	Give the next cohort of patients the dose closest to the ED of interest (ED_95_ or ED_90_) if the *a posteriori* probability for this dose is equal or higher to the ED of interest (ED_95_ or ED_90_).
**Termination of the CRM**	Stop the CRM when a plateau is reached; that is, when eight consecutive patients have been treated at the same level and the recommended dose level if a subsequent patient accrued would remain the same (*8+1* rule). If no plateau is reached, stop the CRM after the inclusion of 36 patients.

### Random walk for the UDM design and ED_x_ determination

Following a suggestion in the literature,^9^ 40 patients will be used to determine the ED_50_ as follows: a random walk using the probability of success of the corresponding dose-response function will be created with the following rule: if a success at dose D is observed for the observation X, the next dose assigned to observation X+1 is dose D−1. On the contrary, if a failure is observed at dose D for the observation X, the next dose assigned to observation X+1 is dose D+1. The ED of interest (ED_95_ or ED_90_) will be estimated via centred isotonic regression by means of the doseFind function of the cir R package^16^ and 50 patients will be recruited to estimate the ED_95_ and ED_90_, respectively. A one-sample proportion test with confidence intervals will be drawn on the last data.^17^

### Random walk for the BCD design and ED_x_ determination

A random walk using the probability of success of the corresponding dose-response function will be used as follows: if a failure is observed for observation X at dose D, an increasing dose (D+1) will be given to the next observation (X+1). On the contrary, if a success is observed for observation X at dose D, a same dose will be given to the next observation in 90% (for the ED_90_) or in 95% (for the ED_95_) of the cases, using a random binomial value. Based on recent research,^18^ 60 observations will be generated to estimate the ED_90_ and 100 observations to estimate the ED_95_. The ED of interest will be estimated through centred isotonic regression by means of the doseFind function of the cir R package.^16^

### Random walks for the CRM design and stopping rules for ED_x_ determination

#### Working model, maximum sample size, and cohort size

For the CRM design, the same working model will be used (prior distribution of 50%, 60%, 75%, 90%, 95%, and 98% for the ED_90_, and 50%, 75%, 90%, 95%, 98%, and 99% for the ED_95_). The working models for the CRM will include theoretical doses <1 and >25, doses <1 being associated with a probability of success of 0 and those >25 with a probability of success of 1, allowing the doses 1 and 25 to be associated with the ED of interest. The targeted dose will always be associated with the ED of interest (ED_95_ or ED_90_) and 36 observations by CRM trial will be performed with cohort size set at two or four observations.

#### Random walk to stop the trial earlier when the tested doses are far from the ED of interest

The random walk for CRM is based on a simple idea: to stop the current CRM when its estimated dose is far (too high or too low, respectively) from the ED of interest. A set of rules based on the ‘*a posteriori*’ distribution probabilities and the mean successes observed were developed and are presented in Appendix 2.

#### Rule to stop the trial earlier when a good candidate for the ED of interest has been found

The trial will be allowed to stop earlier, suggesting the ED of interest has been found, when a plateau has been reached, that is, when eight consecutive patients have been treated at the same level and the recommended dose level if a subsequent patient accrued would remain the same (*8+1* rule).^19^ When a plateau has not been reached, the trial will be stopped after 36 observations have been collected.

## Results

### Estimation of ED_95_

**Fig 2:**
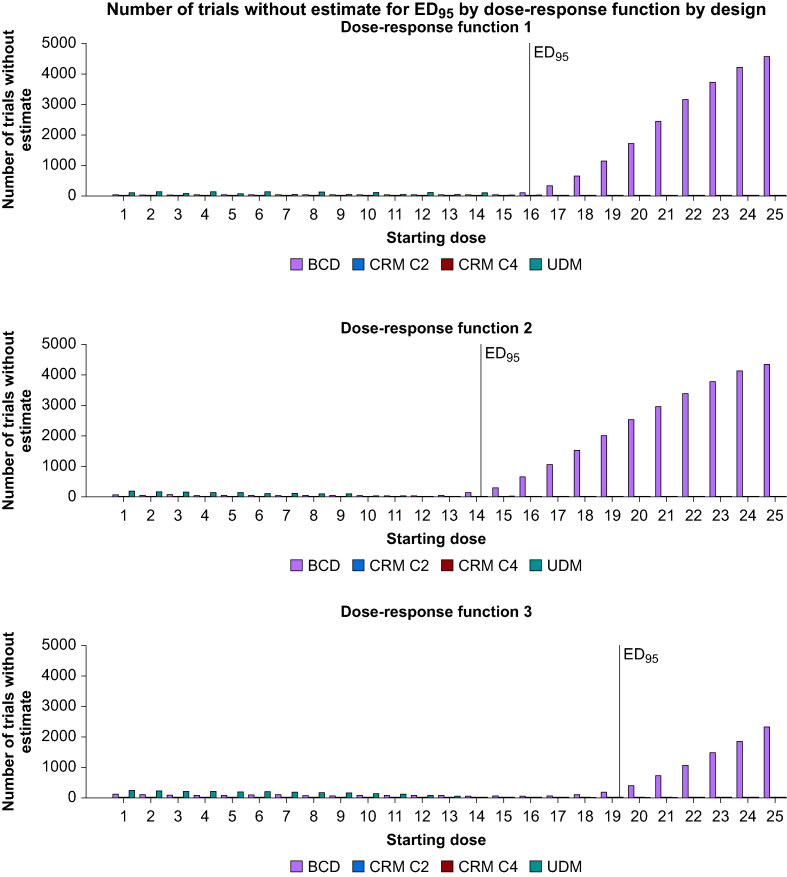
Number of simulations without estimation for ED_95_. BCD, biased-coin up and down; CRM, continual reassessment method; UDM, up and down method.

As indicated in Fig 2, the CRM always finds an estimate for ED_95_ contrary to UDM and BCD. For UDM, starting doses under the ED_95_ are at risk of not finding an estimator for ED_95_. For BCD, all starting doses are at risk of not finding an estimator for ED_95_ and this proportion increases drastically for starting doses greater or equal to ED_95_ minus one dose, with, for some starting doses, between 50% and 90% of the simulations being unable to find an ED_95_.

**Fig 3:**
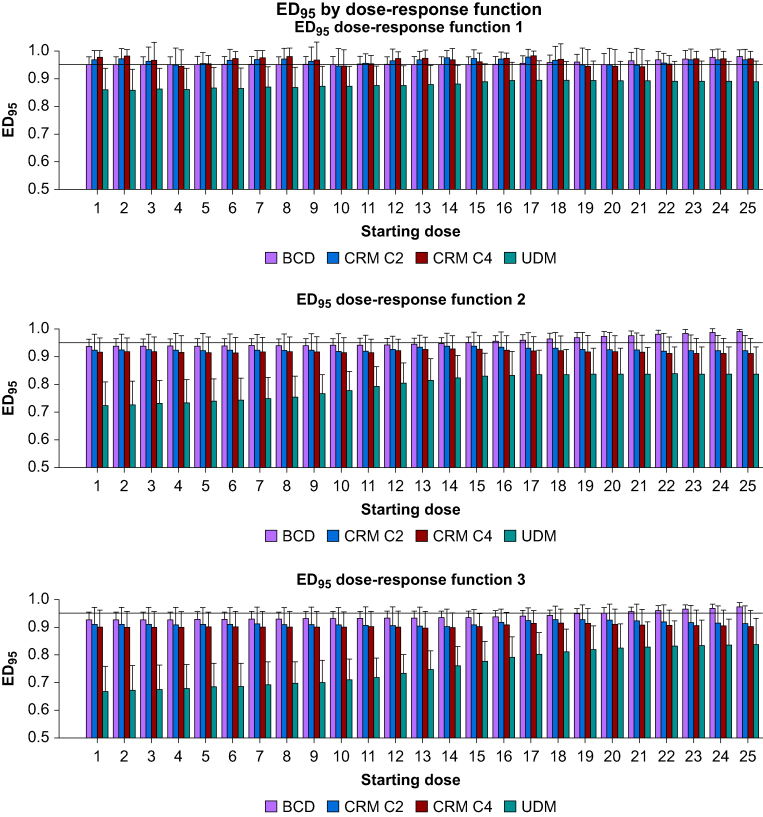
Mean (standard deviation) of the estimated dose for the ED_95_. BCD, biased-coin up and down; CRM, continual reassessment method; UDM, up and down method.

Figure 3 indicates that, when an estimator for the ED_95_ is found, the UDM underestimates it for the three dose-response functions. The BCD is close to the ED_95_ for function 1 for starting doses under and around the true ED_95_, but overestimates the ED_95_ for starting doses above the ED_95_. For functions 2 and 3, the BCD underestimates (2–3%) the ED_95_, and, at higher starting doses, overestimates the ED_95_. For function 2, the two CRM random walks of cohorts of two or four patients are close or slightly overestimate the ED_95_. For functions 3 and 4, CRM with cohorts of four patients underestimate the ED_95_ by 2–5%, respectively; cohorts of two patients being closer to the ED_95_ than cohorts of four patients.

Similar results were observed when the targeted ED was ED_90_ (see results in Appendix 2).

### Number of observations needed and number of failures

**Fig 4:**
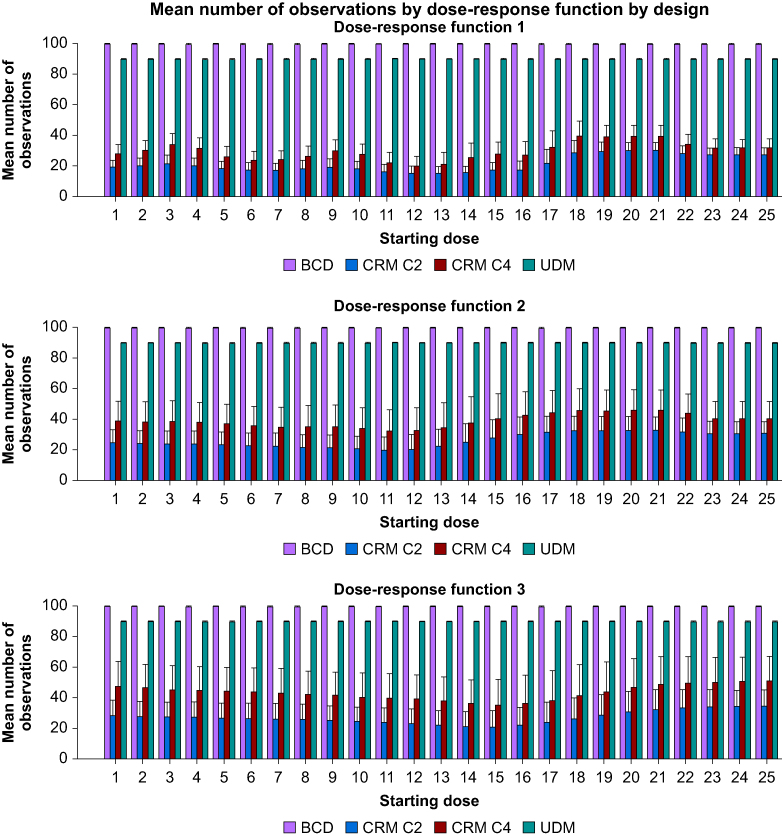
Mean (standard deviation) total number of patients needed to find an ED_95_. BCD, biased-coin up and down; CRM, continual reassessment method; UDM, up and down method.

As indicated in Fig 4, estimating the ED_95_ with the BCD needs 100 observations whereas the UDM requires 90 observations. For the three dose-response functions, CRM requires between 20 and 50 observations on average to estimate ED_95_. CRM with cohorts of two patients requires between 33% and 66% less observations than CRM with cohorts of four patients.

**Fig 5:**
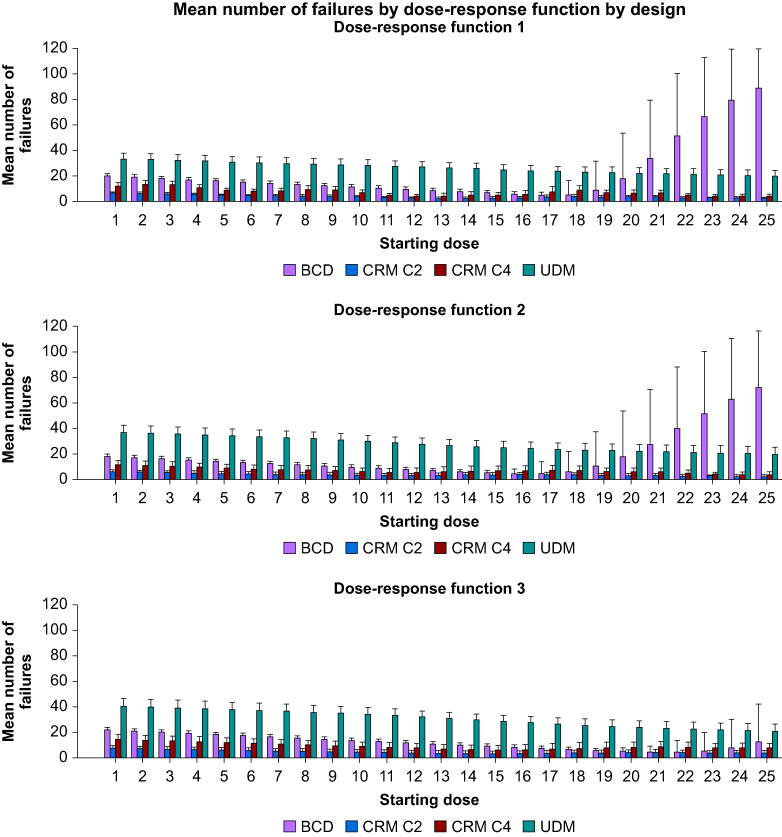
Mean (standard deviation) number of failures for ED_95_. BCD, biased-coin up and down; CRM, continual reassessment method; UDM, up and down method.

Figure 5 presents the number of failures when the estimated ED_95_ is found. It indicates that, compared with UDM and BCD, the CRM has the lowest mean number of failures for the three dose-response functions. It is noteworthy that UDM has a higher number of failures (>30) for the lowest starting doses and that this number decreases as the starting dose increases. The number of failures for BCD exhibits a ‘U-shape’ relationship: starting dose 1 provides around 20 failures, then, after a decrease, the number of failures increases again for higher starting doses. For the three dose-response functions, CRM with cohorts of two and four patients generate six and 13 failures, respectively. This number of failures decreases as the starting dose increases. Similar results are observed when the ED_90_ is targeted (see results in Appendix 2).

## Discussion

This study compared three dose-finding designs (UDM, BCD, and CRM) with a large set of doses (25 doses), replicating clinical research protocols in anaesthesia, and requiring a random walk for CRM. Previous studies have compared dose-finding designs on a limited set of doses^11^^,^^20^ although, in real phase I dose-finding designs, up to 15 doses can be explored.

First, our results indicate that CRM always finds an estimate for ED_95_ and ED_90_, unlike BCD and UDM. Starting with too high a dose for BCD led to inaccurate estimates or non-existing estimates for both ED_95_ and ED_90_. The UDM gives inaccurate results in all cases and does not allow estimation of the ED of interest when starting at doses comprised between dose 1 and two doses under the ED of interest. The CRM gives similar results for all starting doses, but the estimates for ED_95_ are slightly less accurate than BCD estimates for lower starting doses. Compared with BCD, CRM with two-patient cohorts also overestimates ED_90_ for function 1 but provides a better estimation of ED_90_ for functions 2 and 3.

Second, the number of observations and the number of failures associated with the estimate of the ED of interest are always lower with CRM compared with UDM and BCD. Using CRM allows the recruitment of between 20% and 60% fewer patients than with BCD or UDM.

This work shows that CRM better detects the ED_95_ and ED_90_ than BCD under two conditions which have not yet been reported in the literature, as indicated in Table 1: 1) CRM with two-patient cohorts provides more accurate results, needs fewer patients, and is associated with fewer failures for ED_95_ and ED_90_; 2) the dose allocation rule targets the value closest to the ED of interest when the ‘*a posteriori*’ probability value of the recommended dose for the next cohort of patients is greater or equal to the ED of interest. Indeed, when a failure is observed for a cohort of patients, a higher dose is likely to be recommended for the next cohort. This leads to higher doses, especially for two-patient cohorts, as the ‘*a posteriori*’ distribution is less stable with two patients compared with four.^21^ Combined, with the dose-allocation rule we followed, this led the simulations to higher doses. In oncology, the field for which the CRM was originally developed, this dose-allocation rule could lead to overdosing and be extremely dangerous for patients. Therefore, some CRM designs have been specifically developed to avoid this deleterious effect.^22^ In anaesthesiology, drugs often have a shorter half-life and fewer side-effects than in oncology. Thus, such a strategy could be recommended, because overdosing starts when >10% of the target dose is given.^23^

Our results are in agreement with those of other studies comparing UDM and CRM for lower targeted ED^7^^,^^8^ or when the starting dose is not far from the ED of interest in anaesthesiology.^11^ A recent study^11^ reported poor estimation by the CRM when the starting dose is far from the ED of interest. In contrast, the ED estimates in this study with Monte Carlo simulations are rather stable through the different starting doses. This indicates the usefulness of a random walk definition, as was inspired by Kant and colleagues.^24^

Last, our results indicate that the ED of interest is more easily reached when the slope of the dose-response curve is steep. When the shape of the curve is less steep, however, all methods fail to detect the true ED_95_ and only the CRM is able to find the ED_90_.

### Limitations

A first limitation of this study lies in the definition of the random walk for the CRM. The cut-off established on the posterior probabilities and the number of incremental/decremental doses between two CRM were user defined, and other sets of rules may be found. However, if a random walk is possible on the three dose-response functions, alternative random walks could also be built. Our global idea of using a random walk, that is, when a dose-finding design fails to find a dose associated with the ED_95_, is to start with a new CRM at a higher or lower dose. With such an approach, even with a second or a third CRM, a lower number of observations and failures will be reported when determining the ED_95_ or ED_90_ compared with BCD.

A second limitation of the study is the high number of doses tested for each dose-response function. In our study, the number of doses defining the dose-response functions was arbitrarily chosen to be very high, as this will ensure that investigators will reach the ED_95_ or ED_90_ with a smaller number of doses and applying a random walk if necessary.

Last, no sensitivity analysis has been performed with the BCD design. When no estimator for ED_95_ or ED_90_ is found, it indicates a violation of the monotonicity hypothesis, which could be solved by adding more observations at higher doses. However, adding observations to the BCD design will increase significantly the number of observations, and, possibly, the number of failures, compared with the CRM design.

## Conclusions and recommendations

CRM is a valid concurrent dose-finding method to BCD. Indeed, CRM, unlike BCD, is always able to find an estimate for the ED of interest. In addition, CRM requires half as many patients as BCD and produces fewer failures than BCD. Based on our simulations, investigators should avoid using UDM because it always underestimates the ED of interest. CRM should be preferred over BCD when planning a dose-finding trial.

## Authors’ contributions

Statistical analysis and Monte Carlo simulations: JFF

Study design: JFF, PVdL

Interpretation of the data, drafting the article, final approval of the version submitted: all authors

## Declarations of interest

The authors declare that they have no conflicts of interest.

## Funding

Ars Statistica, Nivelles, Belgium.
