# Supplementary material for: A Monte Carlo simulation study comparing the up and down, biased-coin up and down and continual reassessment methods used to estimate an effective dose (ED95 or ED90) in anaesthesiology research
Source: BJA Open. 2023 Sep 27;8:100225. doi: 10.1016/j.bjao.2023.100225 (PMC10542596; doi:10.1016/j.bjao.2023.100225)
Supplement: Multimedia component 1 [file mmc1.docx]

**Appendix 1.**

**Table 1. Dose-Response Functions definition**

| Dose | Probability of success Sigmoid 1 | Probability of success Sigmoid 2 | Probability of success Sigmoid 3 | Probability of success Sigmoid 4 |
| --- | --- | --- | --- | --- |
| 1 | 0,00037266% | 6,94135895% | 6,3225699% | 0,1% |
| 2 | 0,001013% | 10,0134388% | 8,3498711% | 0,25% |
| 3 | 0,00275357% | 14,2371367% | 10,951231% | 0,5% |
| 4 | 0,00748462% | 19,8494209% | 14,23713669% | 1% |
| 5 | 0,0203427% | 26,9781334% | 18,30629% | 2% |
| 6 | 0,05527786% | 35,5320688% | 23,223529% | 3% |
| 7 | 0,15011823% | 45,1222559% | 28,9928305% | 4% |
| 8 | 0,40701377% | 55,0890104% | 35,53206881% | 5% |
| 9 | 1,09880293% | 64,6632087% | 42,6601094361% | 10% |
| 10 | 2,93122308% | 73,1896837% | 50,106692999% | 20% |
| 11 | 7,585818% | 80,2860239% | 57,5485465% | 30% |
| 12 | 18,2440439% | 85,8667649% | 64,66320867% | 40% |
| 13 | 37,7540669% | 90,06321% | 71,182573% | 50% |
| 14 | 62,2482831% | 93,1135739% | 76,92831181% | 60% |
| 15 | 81,7574476% | 95,2766556% | 81,82102415% | 70% |
| 16 | 92,414182% | 96,7837646% | 85,866764899% | 80% |
| 17 | 97,0687769% | 97,8209883% | 89,13172877% | 85% |
| 18 | 98,9013057% | 98,528796% | 91,715216% | 90% |
| 19 | 99,5929862% | 99,0090159% | 93,727800% | 95% |
| 20 | 99,8498818% | 99,3335462% | 95,27665564% | 96% |
| 21 | 99,9447221% | 99,5522789% | 96,4574929% | 97% |
| 22 | 99,9796573% | 99,6994399% | 97,3513273% | 98% |
| 23 | 99,9925154% | 99,7983287% | 98,0242517% | 99% |
| 24 | 99,9972467% | 99,8647258% | 98,52879598% | 99,5% |
| 25 | 99,998987% | 99,9092825% | 98,90593348% | 99,75% |

**Review of the three dose-findings methodologies**

***Up and Down Method (UDM)***

The UDM^1,2^ consists of a phase I clinical trial in two steps.

The first step aims at determining the ED_50_ of the studied anesthesic agent. The researcher starts with a high dose for the first patients and treats the next patient according to the response of the previous one. If this response correponds to a success, the second patient will receive a lower dose. Conversly, if this response corresponds to a failure, the second patient will receive a higher dose. The same dose allocation methodology will be applied to the entire cohort enrolled in the first step of the trial. This part of the trial ends when enough “successes” and “failures” are obtained alternatively, indicating that the ED_50_ can be determined. As suggested by Pace *et al.*, this first step should include at least 20-40 patients to achieve a stable estimate of the target dose^6^. The estimation of the ED_50_ and the ED of choice (usually ED_80_, ED_90_ or ED_95_: called here ED_x_) and their associated 95% confidence interval are obtained by running an isotonic regression or a centered isotonic regression on the data collected.

The second step aims at confirming the ED_x_ determined during the first part of the study. All patients will receive the same dose corresponding to the ED_x_ retained. The number of patients included in the second part of the study is fixed (usually around 40-50) when planning the second phase of the trial.

***Biased-Coin Up and Don Method (BCD)***

The biased-coin Up and Down sequential allocation method^3^ (BCD), also sometimes called up and down sequential allocation method consists of one-step clinical trial, targeting directly the ED of choice^4^. The trial begins with at a dose chosen at random or close to that considered by the clinician to be the ED of interest. The dose administred to each patient is based on the previous’s patient response, conditional to the next algorithm for an ED_90_ or ED_95_: if the dose does not provide adequate analgesia, a higher dose will be given to the next patient. On the other hand, if the dose provided adequate analgesia, the dose administred to the next patient would either be decreased with a probability of 1/10, 1/20, respectively, or would remain the same.

The ED is finally estimated with a linearly interpolated isotonic regression or a centered isotonic regression, which assumes a monotone dose-response relationship: the higher the dose, the higher the success rate. The stopping criterion of the study is set at a predefined percentage (according to the targeted ED: i.e. 90% for ED_90_) of sucessful cases at that dose. A maximum number of patients is often predetermined to avoid oversampling if the boundary could not be met. A recent study recommends to recruit at least 100 and 60 patients to target ED_95_ and ED_90_, respectively^5^.

Mathematically, let *p_k_*, *q_k_*, and *r_k_* denote the probabilities that the treatment dosages will move from level k to *k+1*, don from level *k* to *k-1* and stay at the same level, respectively and *Q(x)* being the response function. The dose-allocation algorithm indicates:

1. when *Q(x)*=0; *pk*, that is, give the next patient a higher dose
2. when *Q(x)*=1; *qk* or *rk*, that is, give the next patient the same dose in 95% of 90% of the cases of decrease one dose in 5% and 10% of the cases for ED_95_ and ED_90_, respectively.

***Continual Reassessment Method (CRM)***

The Continual Reassessment Method (CRM) is a multiple-step(s) clinical trial that uses a bayesian methodology in order to estimate the ED chosen by the researchers. The basic idea of baeysian statistics is to use the knowledge someone has of a certain phenomenon (‘*a priori*’) to model a relationship between variables, with a probability distribution of the a priori (‘*prior distribution*’), which represents the uncertainty about the variable to be estimated (the ED of interest in our case: ED_x_). Using the “*a priori”*, the “*prior distribution”* and the collected data, the algorithm calculates the “*a posteriori”* distribution of the data, that is, the distribution probability of the random variable to be estimated (here, the ED_x_).

The general idea behind the CRM is to treat each patient with the dose most likely to be associated with the target ED_x_^6^. Concretely, the trial starts with the definition of a range of doses to be investigated (usually 6 doses), and assign a theoretical ED to each of these doses (=the working model or in bayesian terms, the ‘a priori’). Once the working model is established and the doses associated with the working model are chosen, the trial could start. A first cohort of patients (typically 2 or 4) is recruited and received the dose expected to be associated with the target ED. After collecting the responses (successes/failures), the CRM algorithm is executed, which produces the ‘a posteriori’ ED for each dose, that is, the best statistical guess of the ED associated with each dose. Following consultation between the statistician and the clinician, the dose to be given to the next cohort is chosen. This consultation is of major importance, especially at the beginning of the trial when the CRM model is built on a few patients^7^. The different steps are then repeated until 40 patients have been recruited, unless:

a) the collected data indicates clearly that it is likely that the ED_x_ will not be found (=when the estimated posterior probability of response is either too low or too high for all dose levels)

b) a suitable estimation of the ED_x_ is obtained, based on the predictive gains (mean and maximum) of further patients’ inclusion on the response probability and on the width of its credibility interval (lower than 5%)^7,8^.

Mathematically, assuming a dose-failure relationship, with patient responding better to higher doses, we want to find the ED_95_ or ED_90_; that is, the dose deﬁned as the 5^th^ or 10^th^ percentile of the dose–failure relationship, which is modelled throughout a power model as follows:

P(Y=1/x_i_) = p_i_^θ^,

where θ is the model parameter to be estimated, considered as a random variable with exponential unit prior, and p_i_ (i = 1, …k) is the initial guess of failure probabilities at the i^th^ dose level.

The CRM is conducted as follows: a ﬁrst cohort of four patients is administered the initial candidate of the ED_95_ or ED_90_. Then, once the response is observed for all patients in the cohort, CRM is applied in order to provide the actualized posterior distribution of the model parameter, from which the posterior mean estimate is computed, E (θ/y). This estimate is used in the above formula, to give an updated probability of the ED probability for each dose level. The dose level closest to the ED_95_ or ED_90_ is chosen for candidate by the CRM, and this dose is given to the next cohort of patients.

**Random walk for the CRM**

**Rules to stop the trial earlier when a dose is far from the ED of interest**

Because the CRM is not used as recommended (starting with the dose thought to be associated with the ED of interest), we developed a random walk to increase or decrease the starting dose of a next CRM if case the current CRM failed. Sigmoid 4 was used to calibrate the random walks and the set of rules when stopping a CRM and defining the starting dose of the next CRM. We tried a lot of random walks and only the random walk giving the best results on sigmoid 4 is used for the three other sigmoid.

As indicated by figure 1, the CRM was allowed to stop earlier in the next cases:

1. when the upper posterior probability is lower or equal to 92.50% or 87.50% for ED_95_ and ED_90_, respectively (suggesting that the targeted doses were too low);
2. when the lower posterior probability is upper or equal to 70% or 75% for ED_95_ and ED_90_, respectively (suggesting that the targeted doses were too high);
3. for the first CRM only, when the observed success probability was above 90% for at least 12 observations, the CRM was stopped, indicating a possible too high starting dose. In this case, the dose given to the next CRM equals the rounding of the starting dose for the first CRM divided by 2 (e.g.: if the starting dose for the first CRM was 23 and a mean success above 90% was observed for at least 12 observations, the second CRM started at dose 12, which rounds 23 divided by 2).

Rules 1 and 2 were based on previous research^8,9^. The added value of the proposed random walks lies the third set of rules: suggesting how many dose increments/decrements should be performed when deciding which first dose should be given to the next CRM trial.

**Figure 1. Global rules established to find an estimator for ED_95_ and ED_90_**


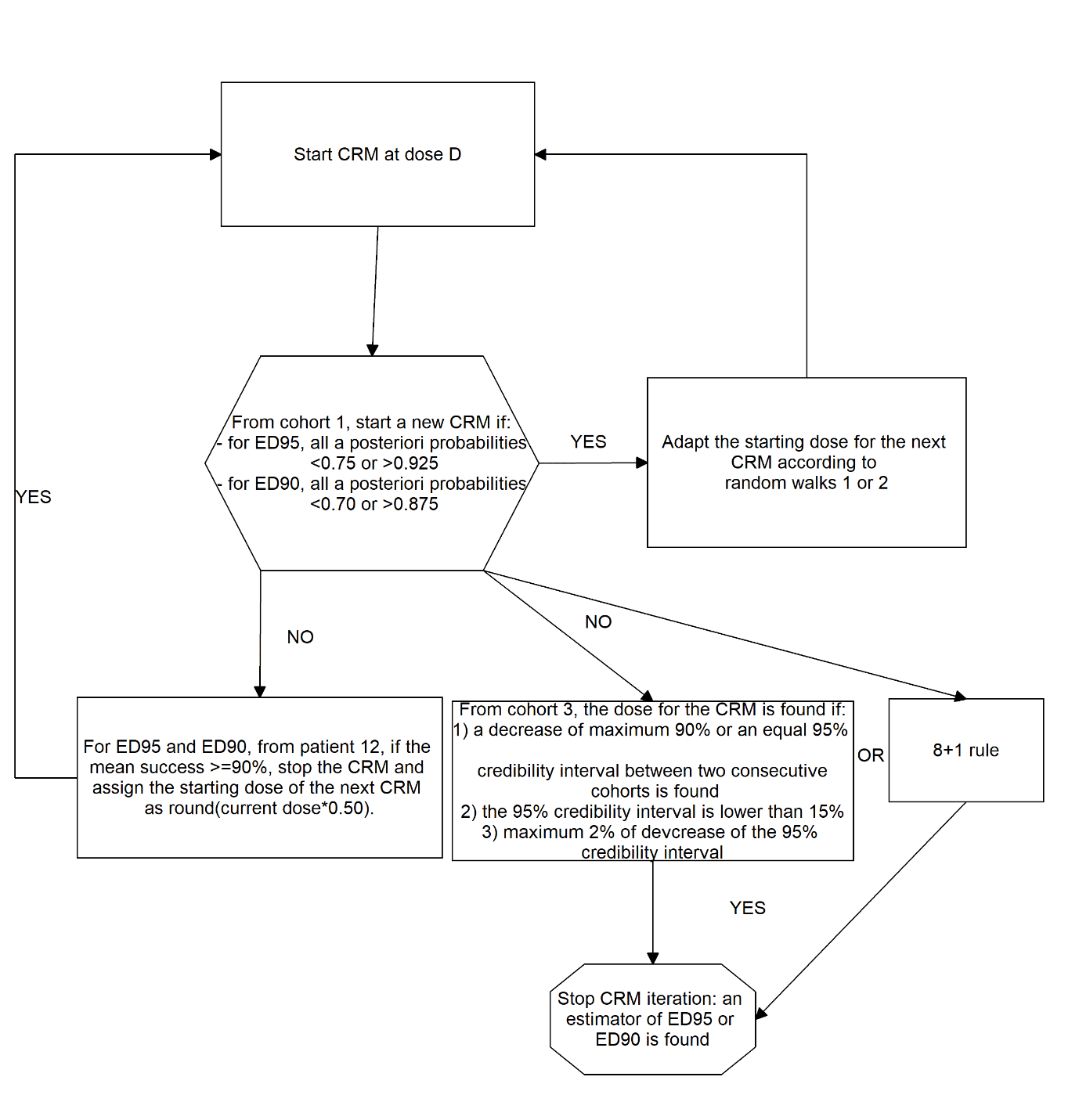


In the other cases, the starting dose for the subsequent CRM were decided based on the percentage of success obtained in the current CRM as indicated by figure 3 for the two random walks. Up to 10 CRM were foreseen to find an estimator for the ED of interest.

**Figure 2. Random walks 1 & 2**


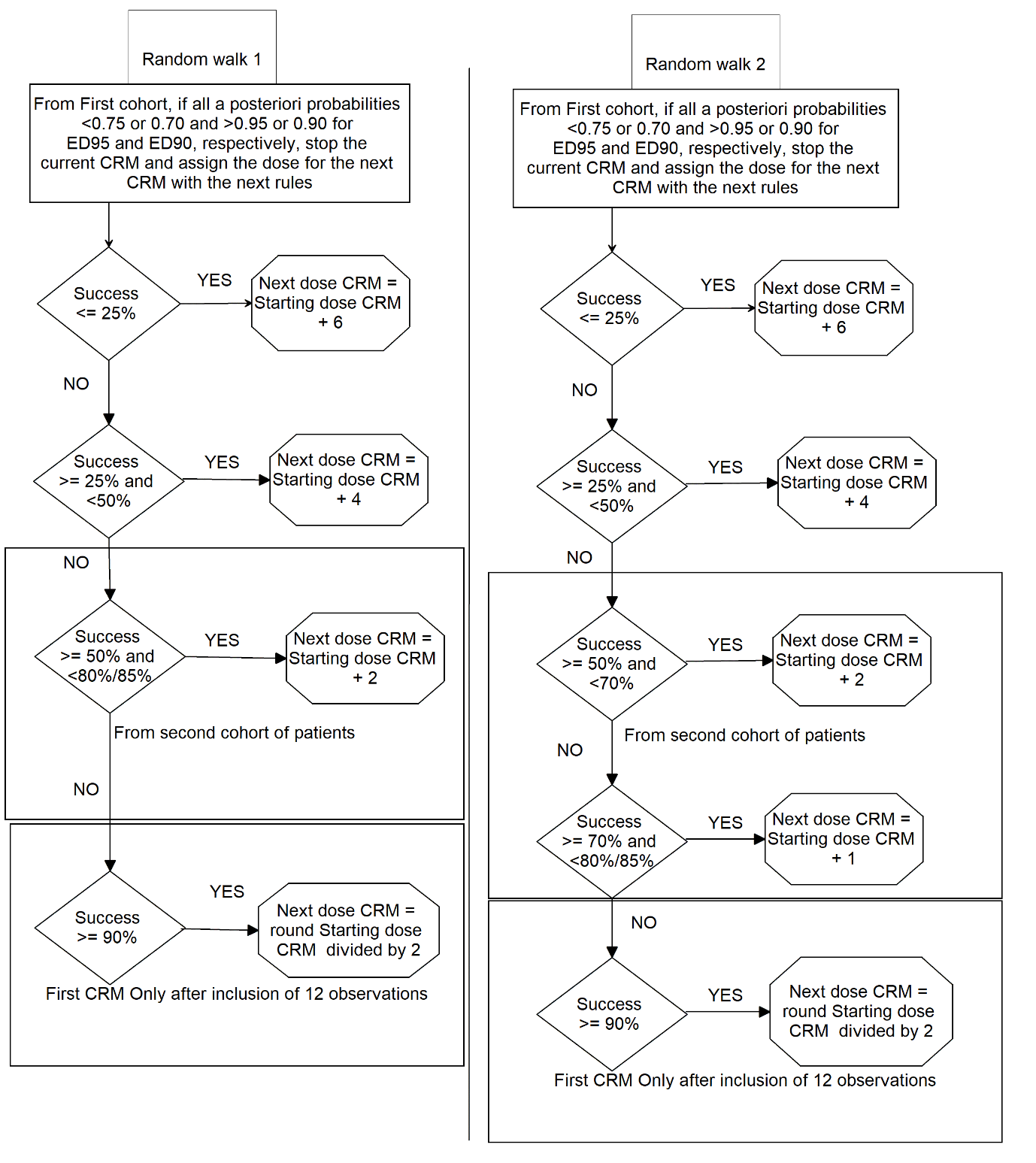


**Rules to stop the trial earlier when a dose is retained to be the ED of interest**

**Rule 1: criteria reached on the credibility interval**

Several stopping rules are available allowing the trial to terminate earlier, when the ED of interest has been identified: 1) when enough precision of the confidence interval/credibility interval has been found^10–12^; 2) when the dose associated with the ED of interest is likely to be the same that the dose found before the end of the trial based on the simulation of inclusion of further patients until the end of the trial^11,12^; 3) when the dose given remains the same for *n* consecutive patients and the next cohort of patients will be given the same dose again (*n+1* or *plateau* *rule*)^13^. Rules based the inclusion of further patients give good results, but are computationally intensive. Our goal was focus on rules directly accessible to statisticians and clinicians performing a CRM: 1) those based on the credibility interval and 2) when a plateau is reached (*n+1* rule). We used sigmoid 1 to test and define a set of rules based on the credibility interval. An estimator for the ED_95_ or ED_90_ was found, which allowed the trial to be terminated earlier, when:

1. the confidence interval of the targeted dose is inferior or equal to 15%, or
2. the decrease in length of the credibility interval between two cohorts is lower than 10 percent, or
3. the decrease in the length of the 95% credibility interval is lower than 2%, suggesting a stabilization of the credibility interval.
4. the a posteriori was within a range of 1.25% around the ED of interest.

The CRM was declared successful when 1) 40 observations were successfully generated without satisfying stopping criteria; or 2) when stopping criteria described above were met. This rule is presented in figure 1.

**Rule 2: a plateau is reached**

Alternatively, we defined a “*8+1”* rule, an alternative to the *6+1* rule^13^ for the study of cohorts of 4 patients: the trial was allowed to stop when 8 consecutive patients received the same dose, and the dose for the next cohort of patients was identical those given to the 8 patients, the CRM reaching a plateau. The CRM was declared successful when 1) 36 observations were successfully generated without satisfying stopping criteria; or when the *8+1* rule was met.

**Comparison of the results of the two rules and the random walks**

The two stopping rules described above allowed an estimation of the ED_95_ and ED_90_ in the 5000 simulations done by CRM. Figures 3 to 20 present the results of the two CRM stopping rules (ED_x_ found, number of observations needed and number of failures). For ED_95_ and ED_90_.

The number of observations and number of failures are rather similar, when comparing the two stopping rules (credibility interval vs *8+1* rule). However, the *8+1* rule is more stable and gives estimates of ED_95_ and ED_90_ which depend less on the starting dose than the stopping rule based on the credibility interval on the three studied sigmoid. Indeed, the estimate of the ED_95_ increases for the three sigmoid as starting dose increases for the stopping rule based on the credibility interval. For ED_90_, a lowest variability around the estimates is found for the *8+1* rule, compared to the rule based on the credibility interval, and the estimates for the *8+1* rule are closest to the ED_90_ for sigmoid 2 and 3. No difference is found between the two random walks for the *8+1* rule, but a difference is found between cohorts of 4 patients and cohorts of 2 patients. The main article presents the results of random walk 1 for the cohorts of 2 and four patients.

**Figure 3. ED_95_ estimation by CRM design and cohort size for dose-response function 1**


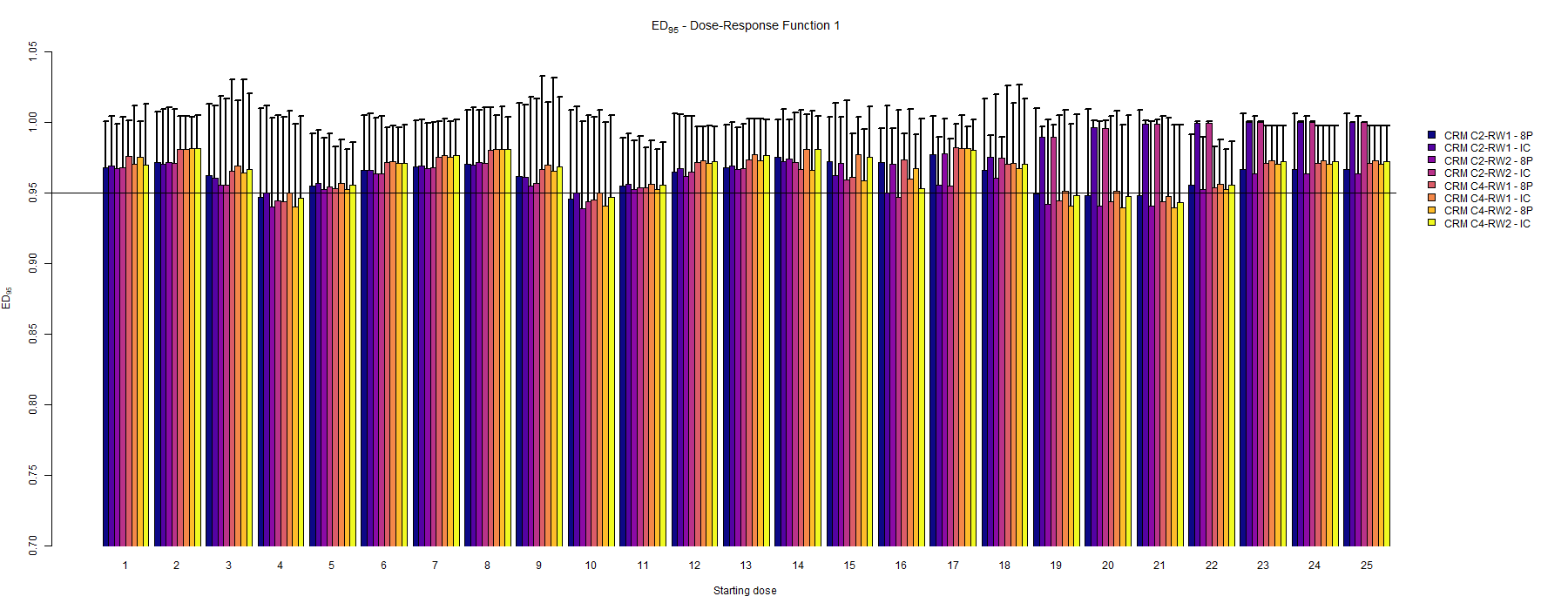


**Figure 4. ED_95_ estimation by CRM design and cohort size for dose-response function 2**


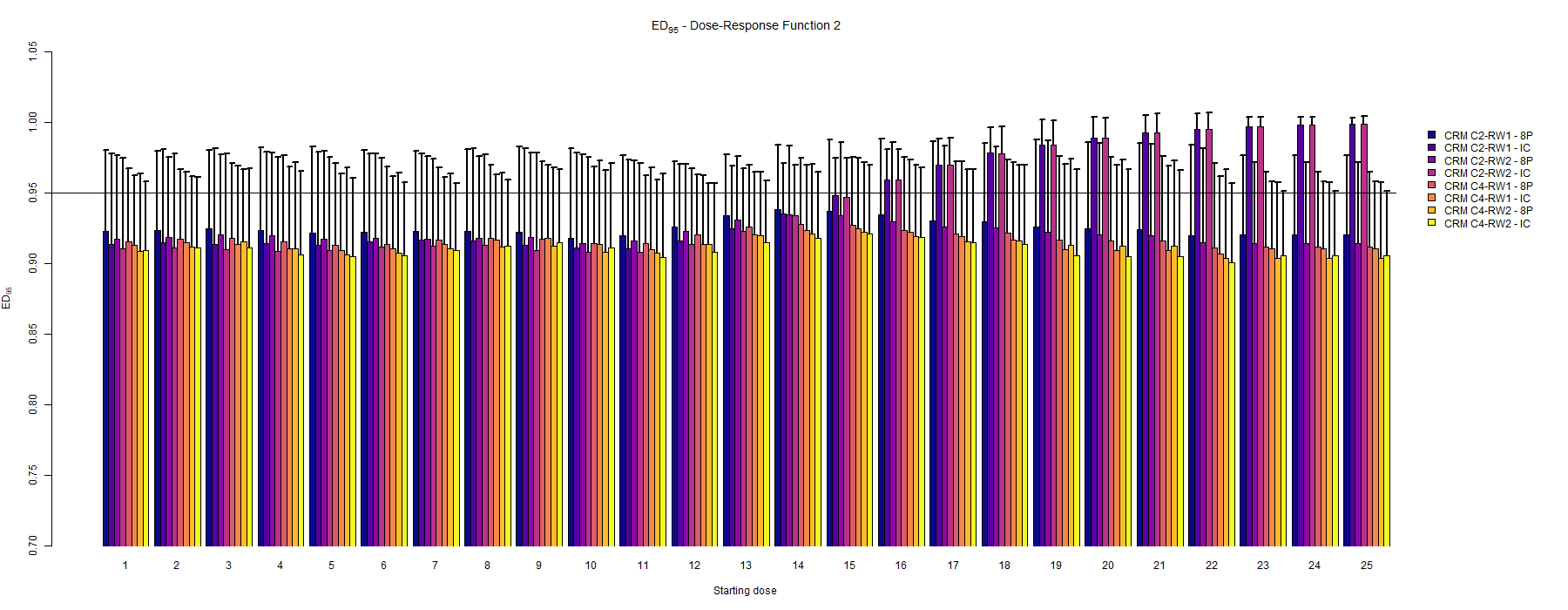


**Figure 5. ED_95_ estimation by CRM design and cohort size for dose-response function 3**


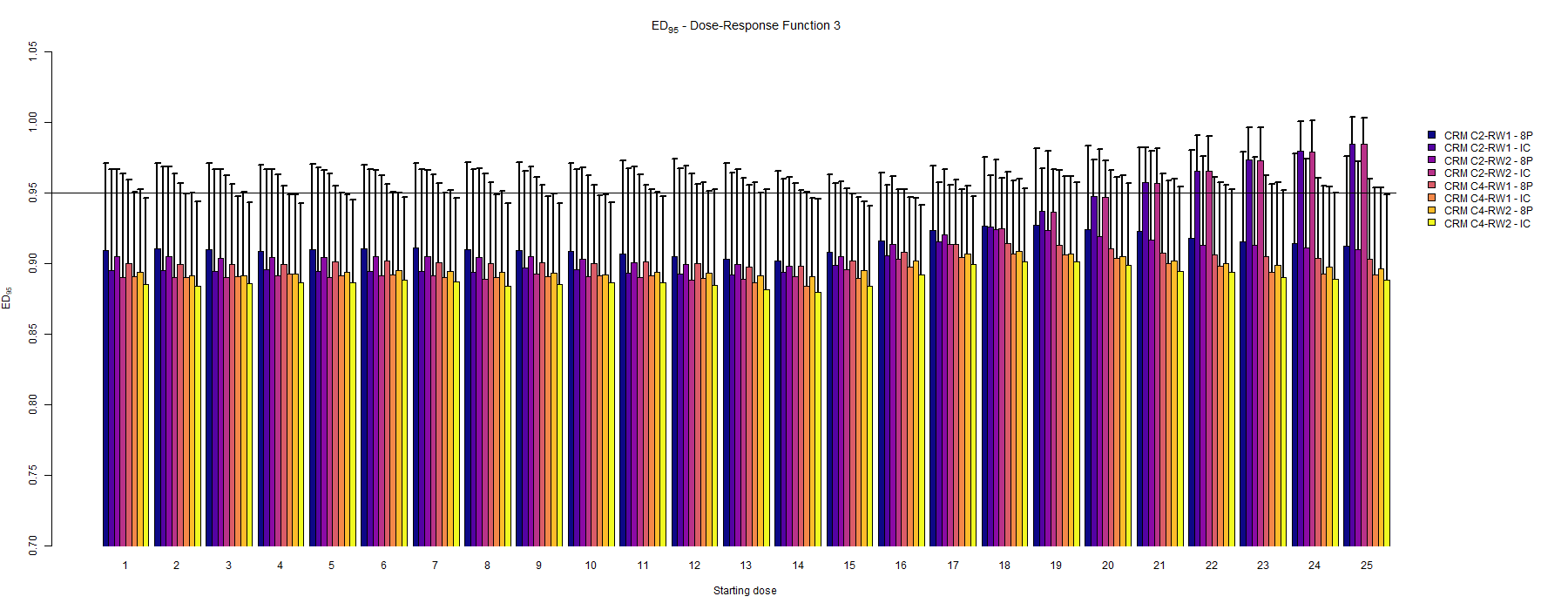


**Figure 6. ED_90_ estimation by CRM design and cohort size for dose-response function 1**


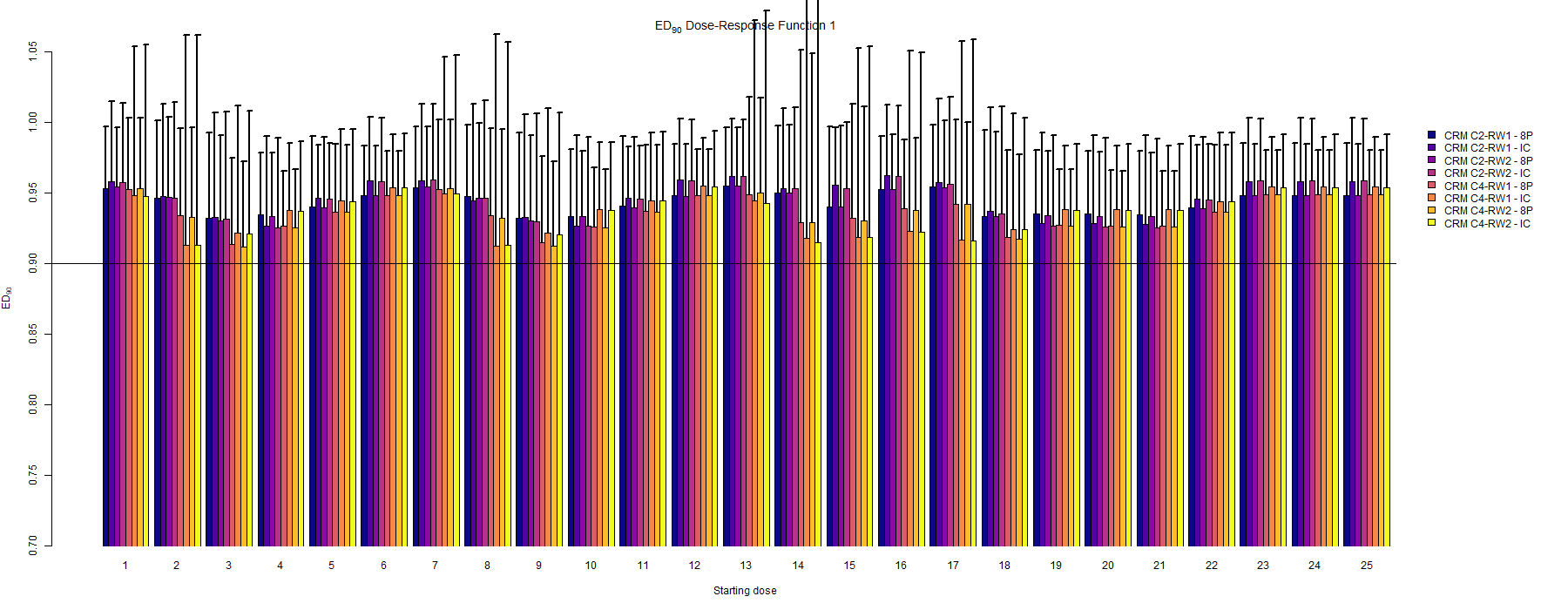


**Figure 7. ED_90_ estimation by CRM design and cohort size for dose-response function 2**


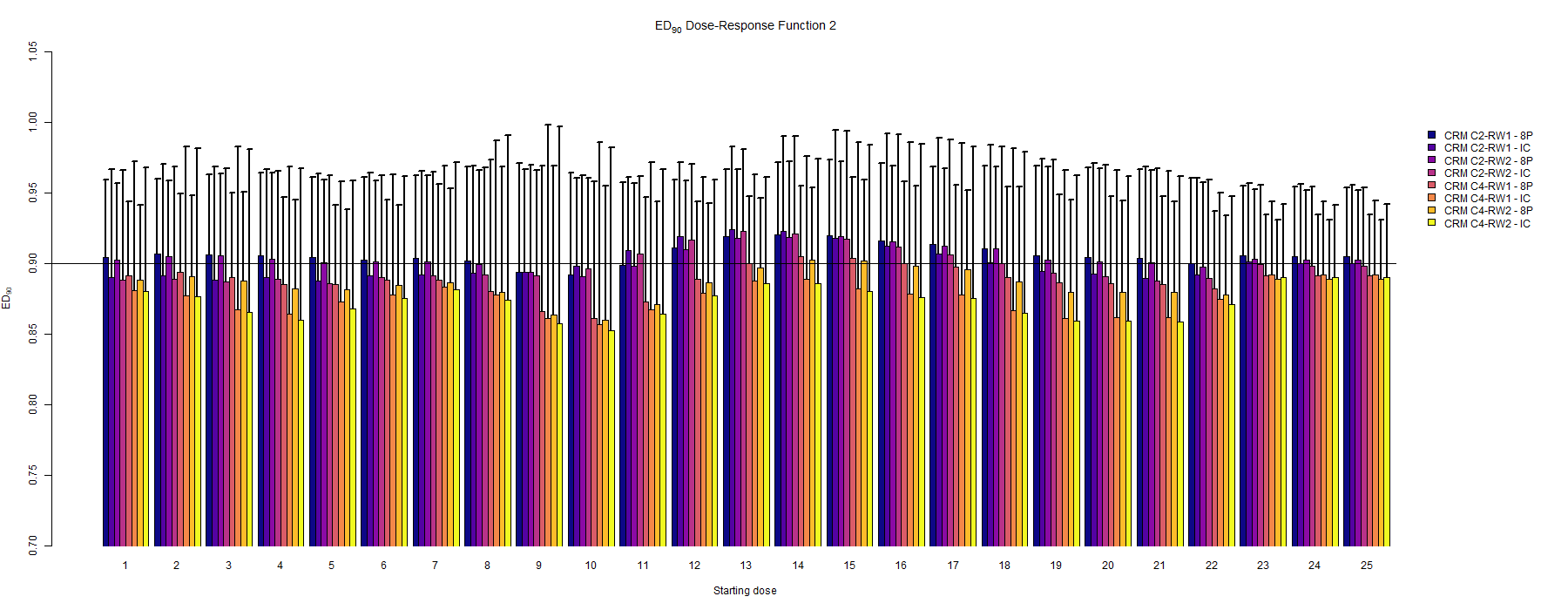


**Figure 8. ED_90_ estimation by CRM design and cohort size for dose-response function 3**


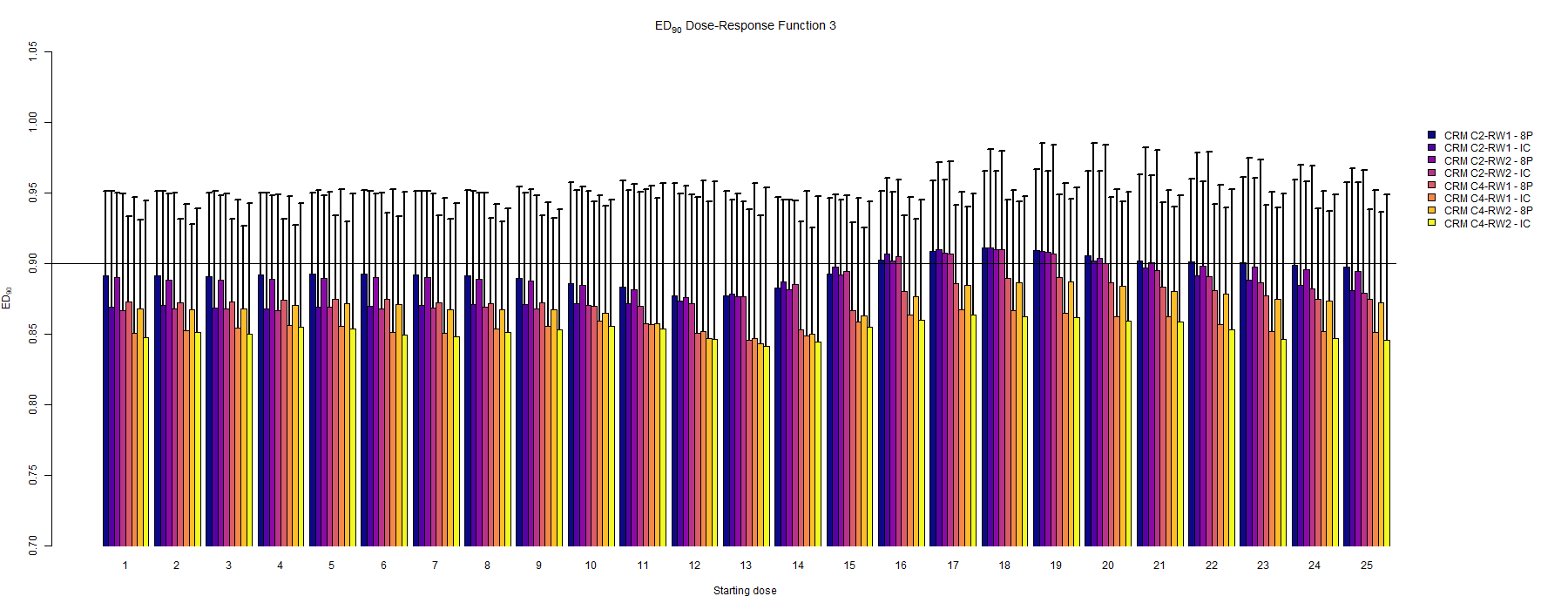


**Figure 9. Mean ± Standard Deviation number of observations for ED_95_ by CRM design and cohort size for dose-response function 1**


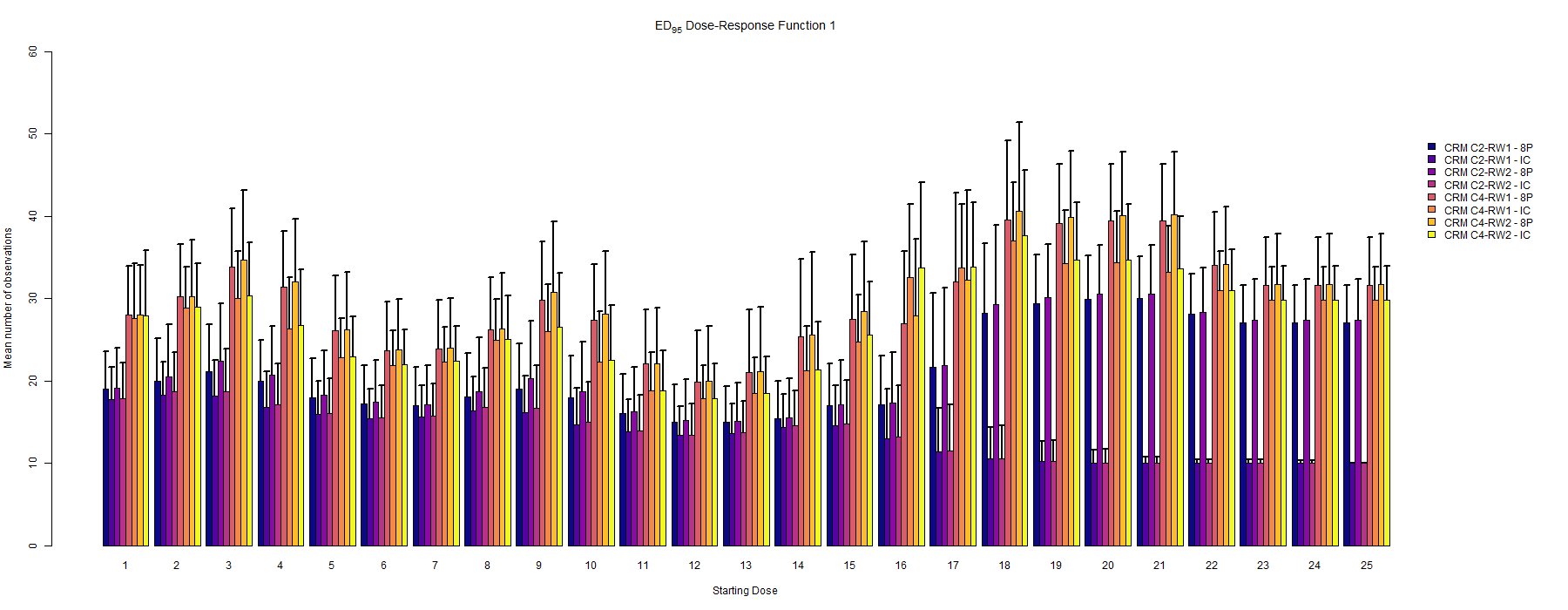


**Figure 10. Mean ± Standard Deviation number of observations for ED_95_ by CRM design and cohort size for dose-response function 2**


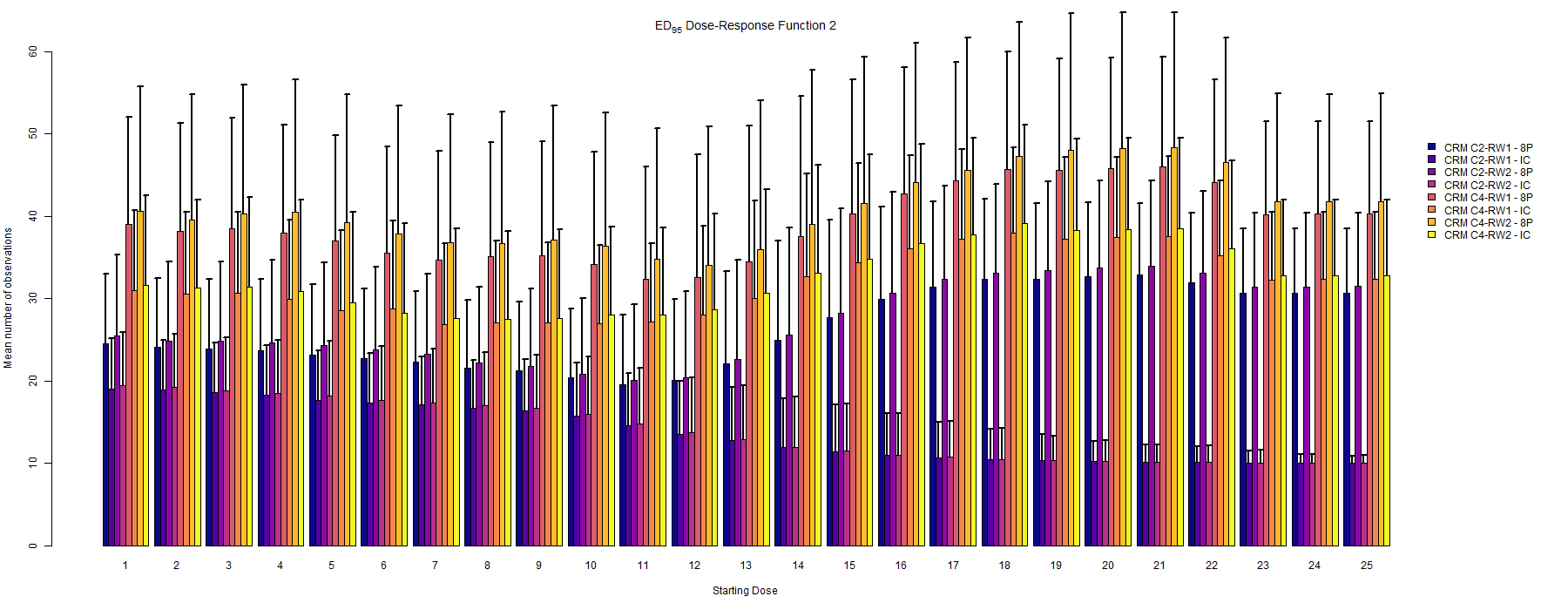


**Figure 11. Mean ± Standard Deviation number of observations for ED_95_ by CRM design and cohort size for dose-response function 3**


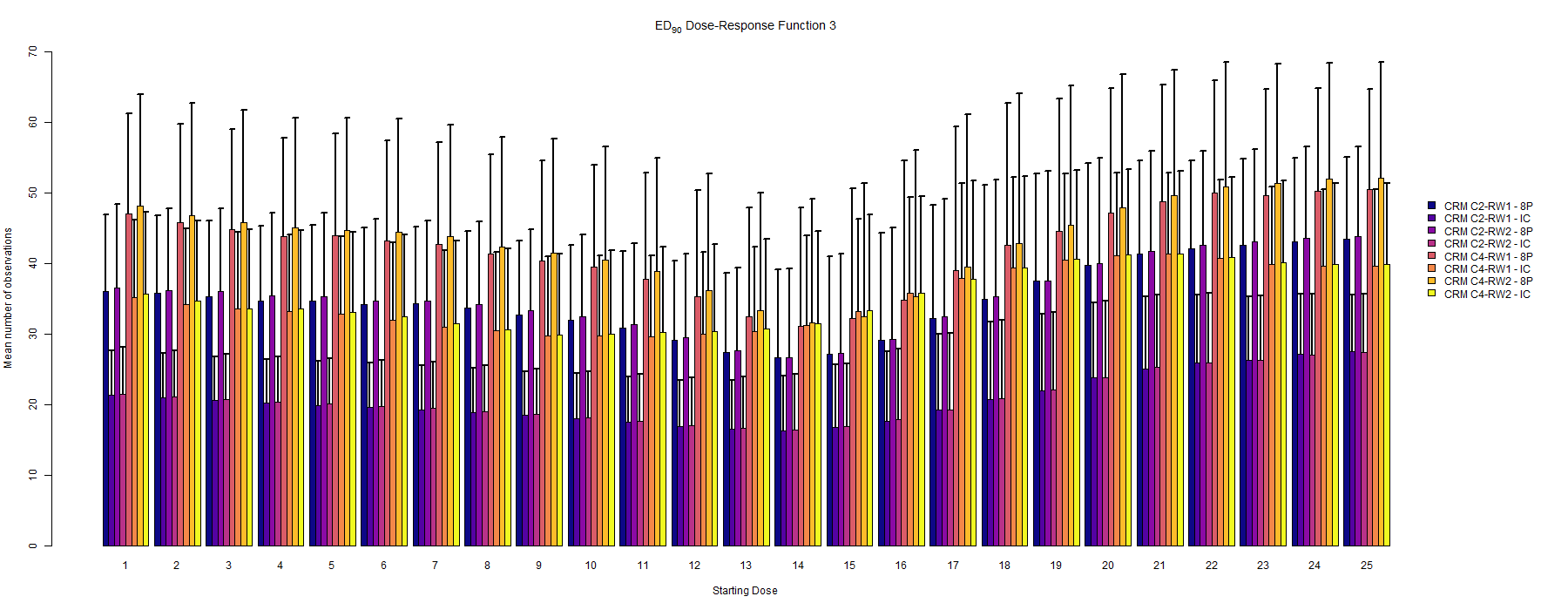


**Figure 12. Mean ± Standard Deviation number of observations for ED_90_ by CRM design and cohort size for dose-response function 1**


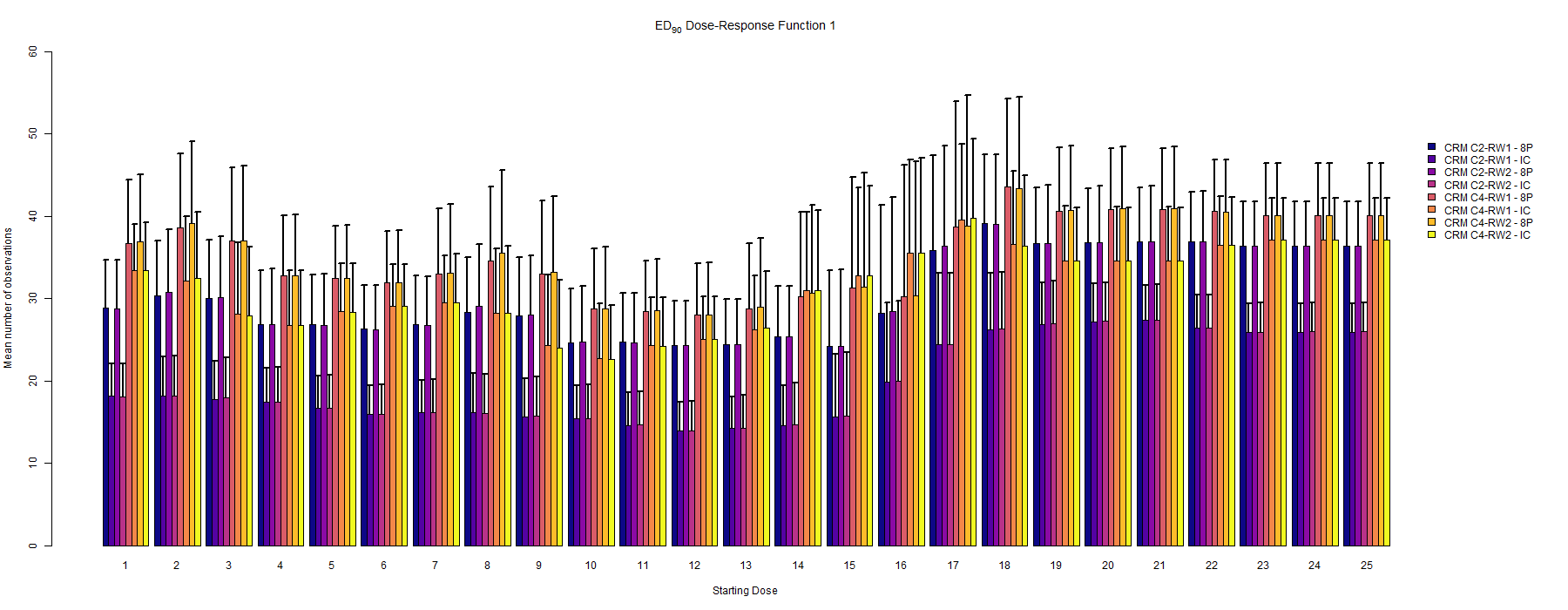


**Figure 13. Mean ± Standard Deviation number of observations for ED_90_ by CRM design and cohort size for dose-response function 2**


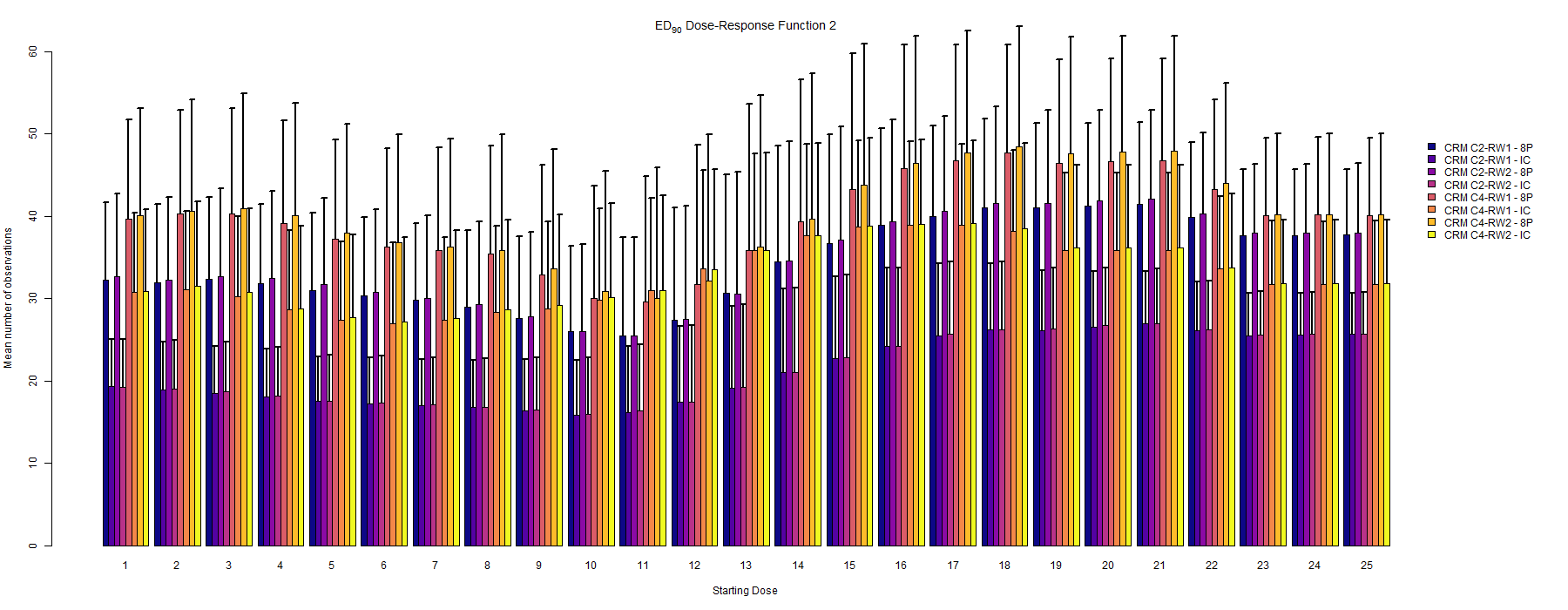


**Figure 14. Mean ± Standard Deviation number of observations for ED_90_ by CRM design and cohort size for dose-response function 3**


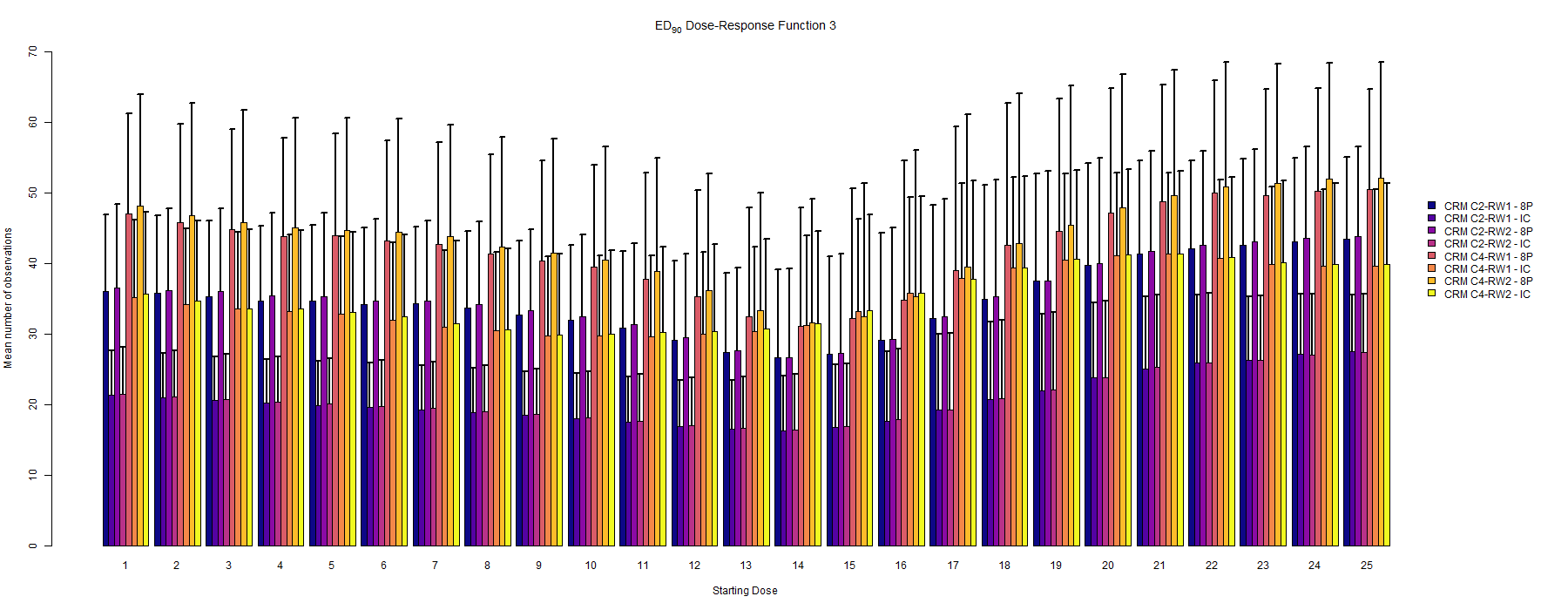


**Figure 15. Mean ± Standard Deviation number of failures for ED_95_ by CRM design and cohort size for dose-response-function 1**


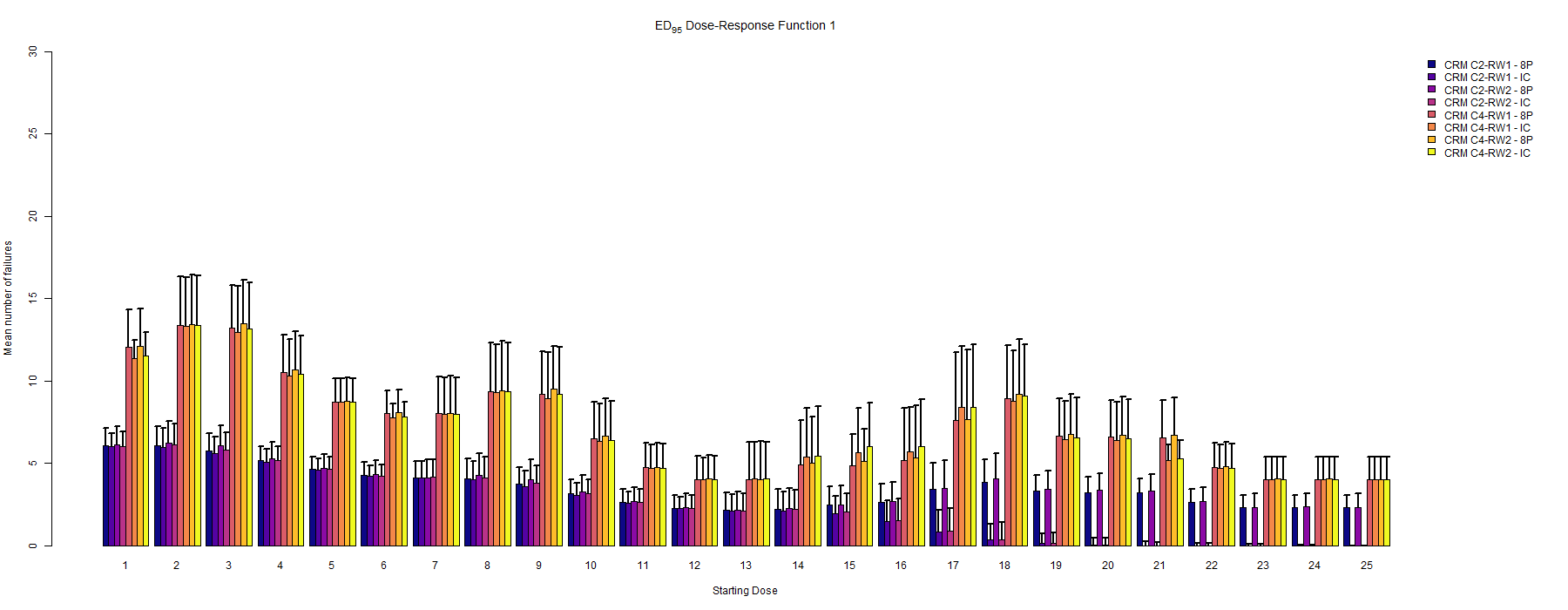


**Figure 16. Mean ± Standard Deviation number of failures for ED_95_ by CRM design and cohort size for dose-response function 2**


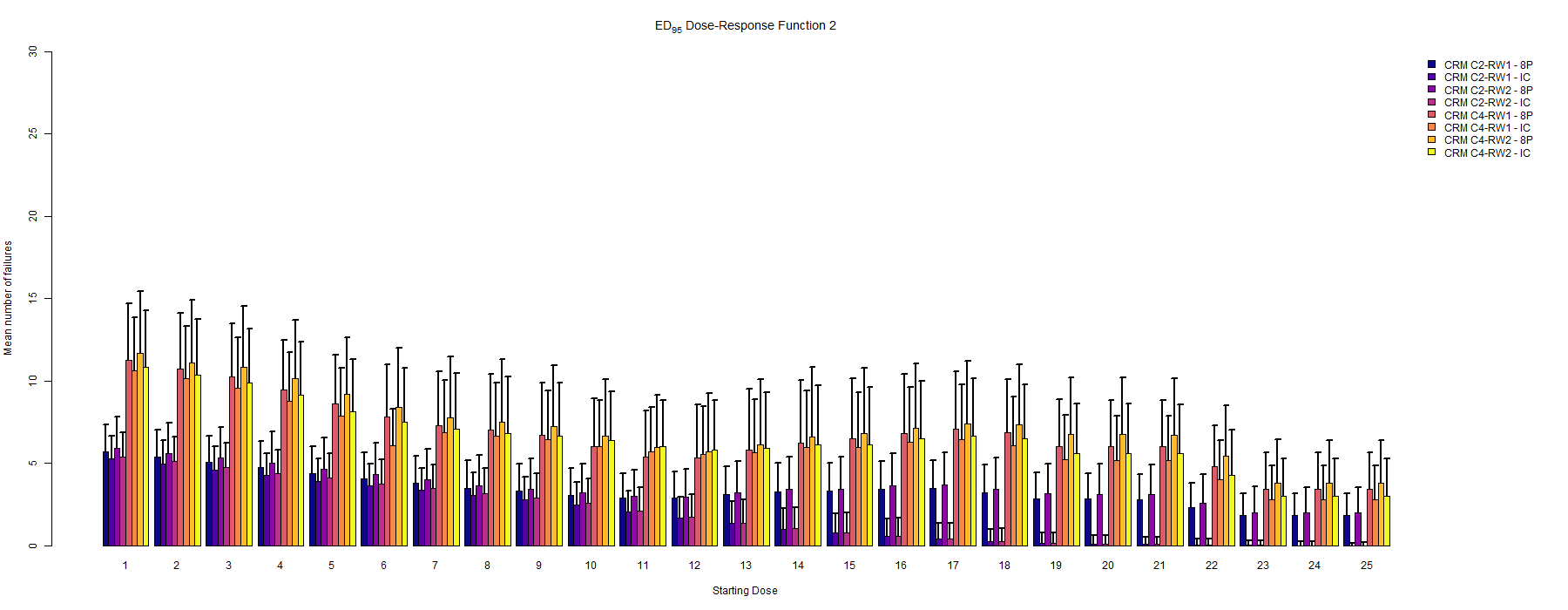


**Figure 17. Mean** **± Standard Deviation number of failures for ED_95_ by CRM design and cohort size for dose-response function 3**


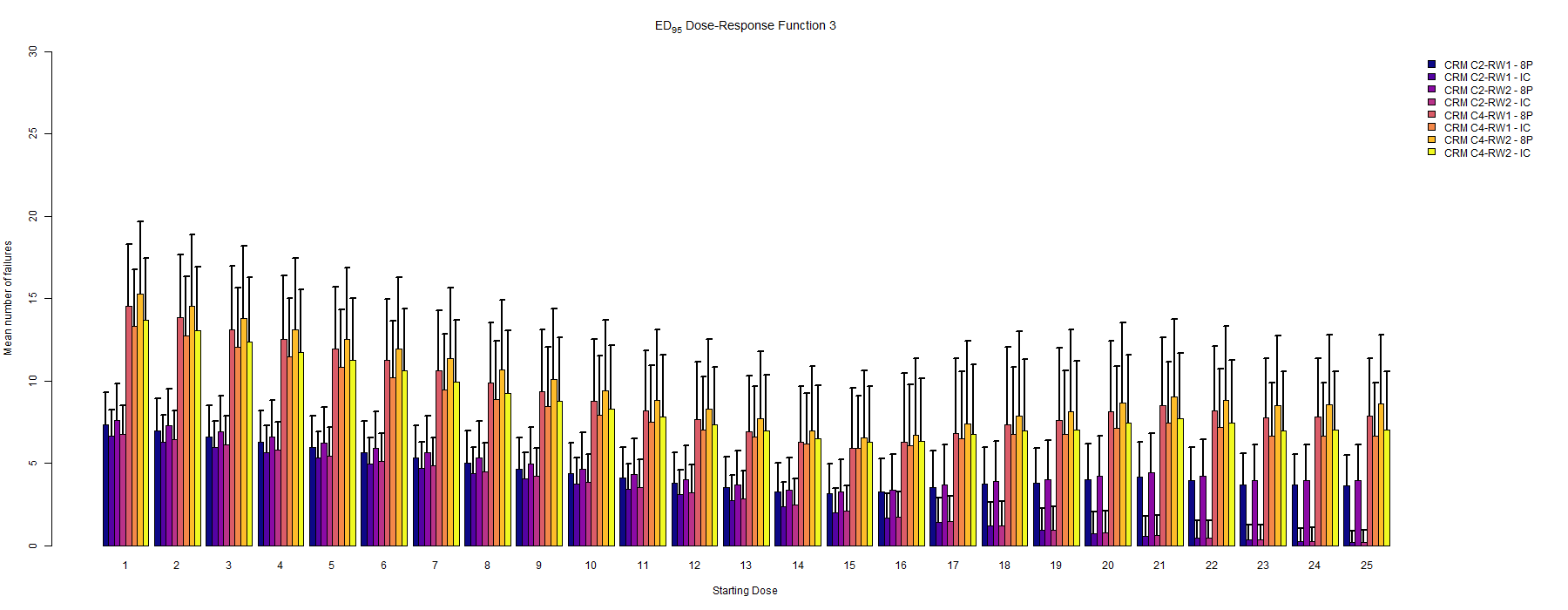


**Figure 18. Mean ± Standard Deviation number of failures for ED_90_ by CRM design and cohort size for dose-response function 1**


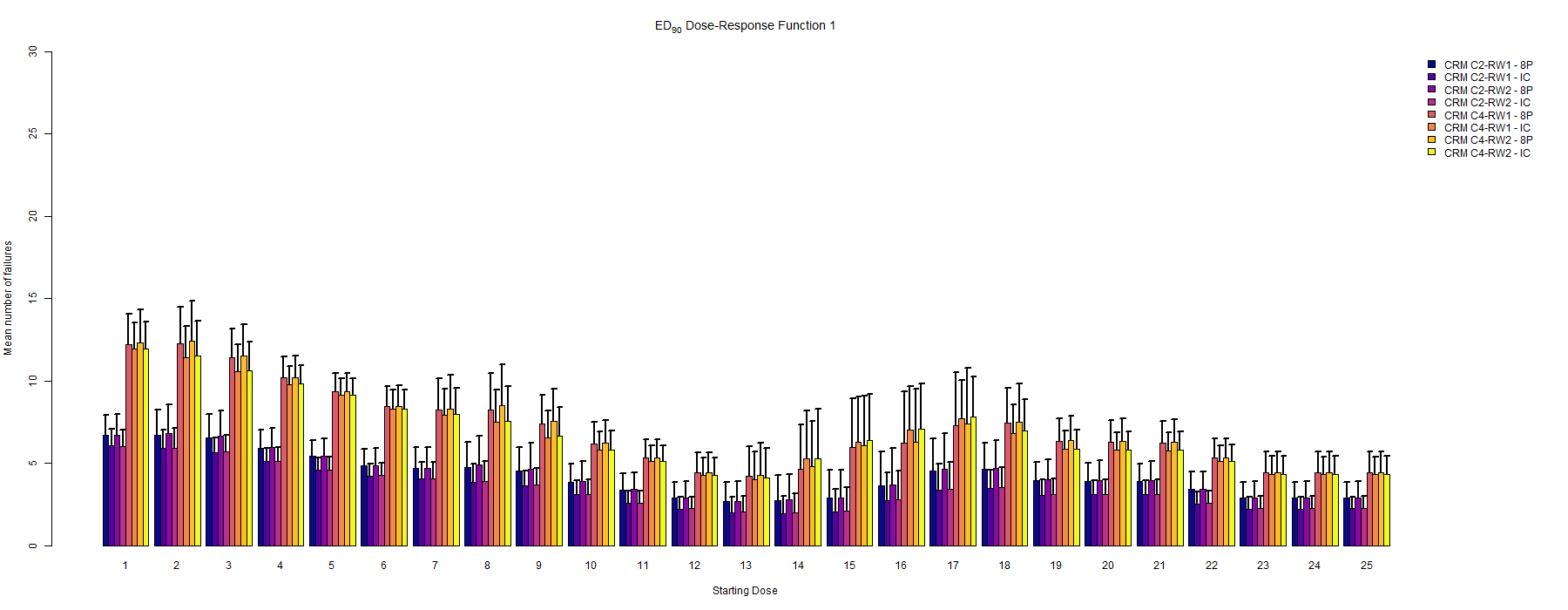


**Figure 19. Mean ± Standard Deviation number of failures for ED_90_ by CRM design and cohort size for dose-response function 2**


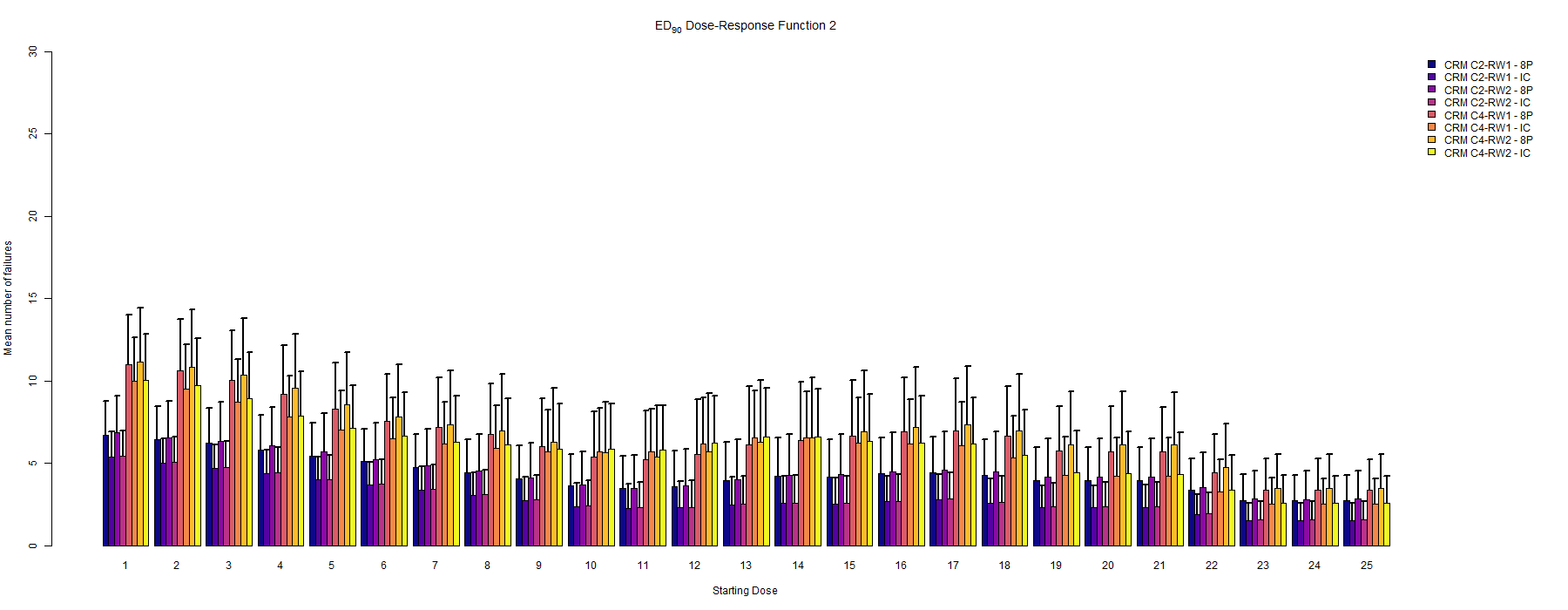


**Figure 20. Mean ± Standard Deviation number of failures for ED_90_ by CRM design and cohort size for dose-response function 3**


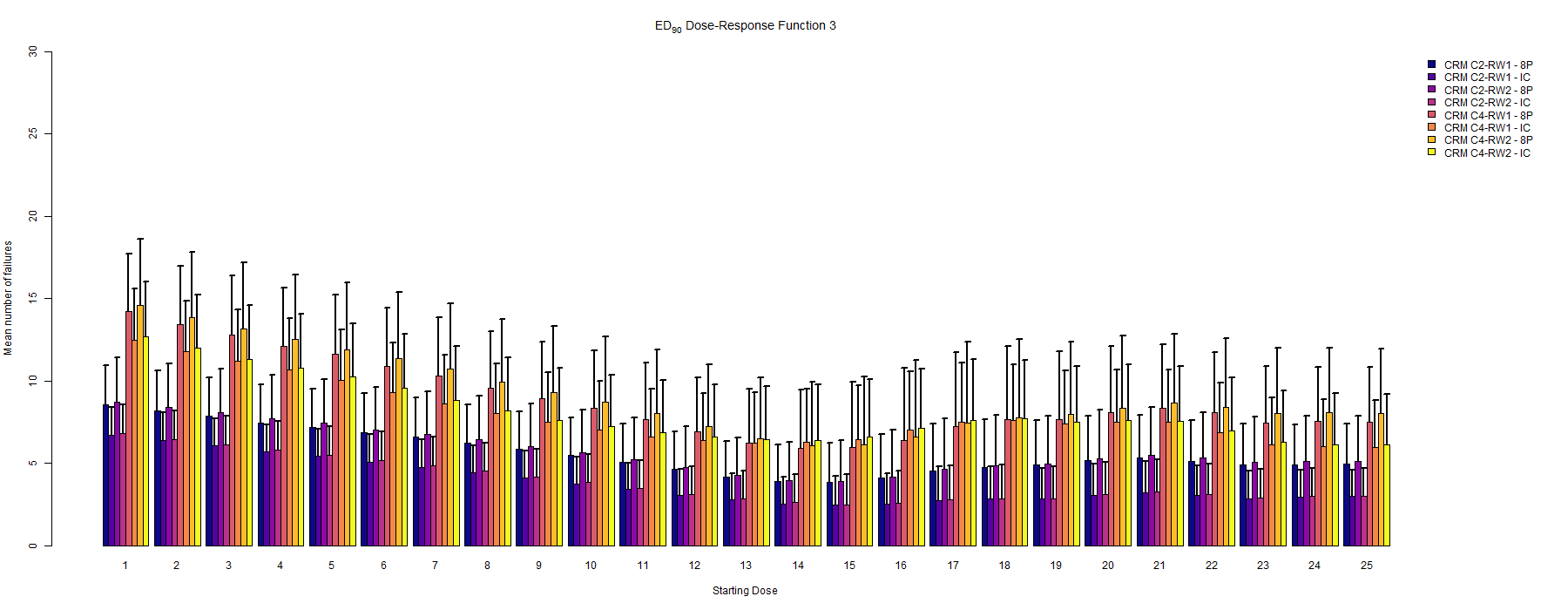


**Results for the ED_90_**

**Figure 21. Number of trials without estimate for ED_90_ by dose-response function and by design**


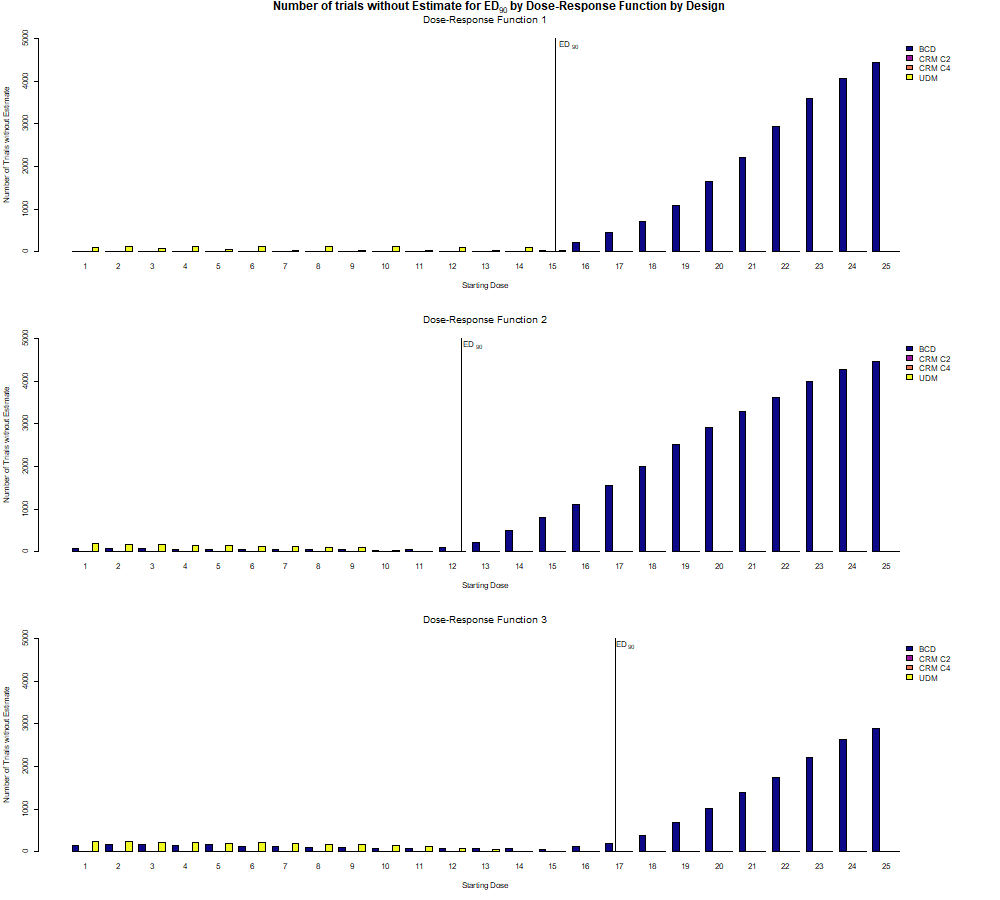


The above figure indicates that all CRM find an estimate for ED_90_, which is not the case for UDM and BCD. For UDM, only starting doses under the ED_90_ are at risk of not finding an estimator for ED_90_. On the contrary, for BCD, all starting doses are at risk of not finding an estimator for ED_90_ and this proportion increases drastically for starting doses greater or equal to one dose under the ED_90_, with, for some starting doses, between 50% and 90% of the simulations unable to find an ED_90_.

**Figure 22. Mean ± Standard deviation of Estimated ED_90_ by dose-response function and by design**


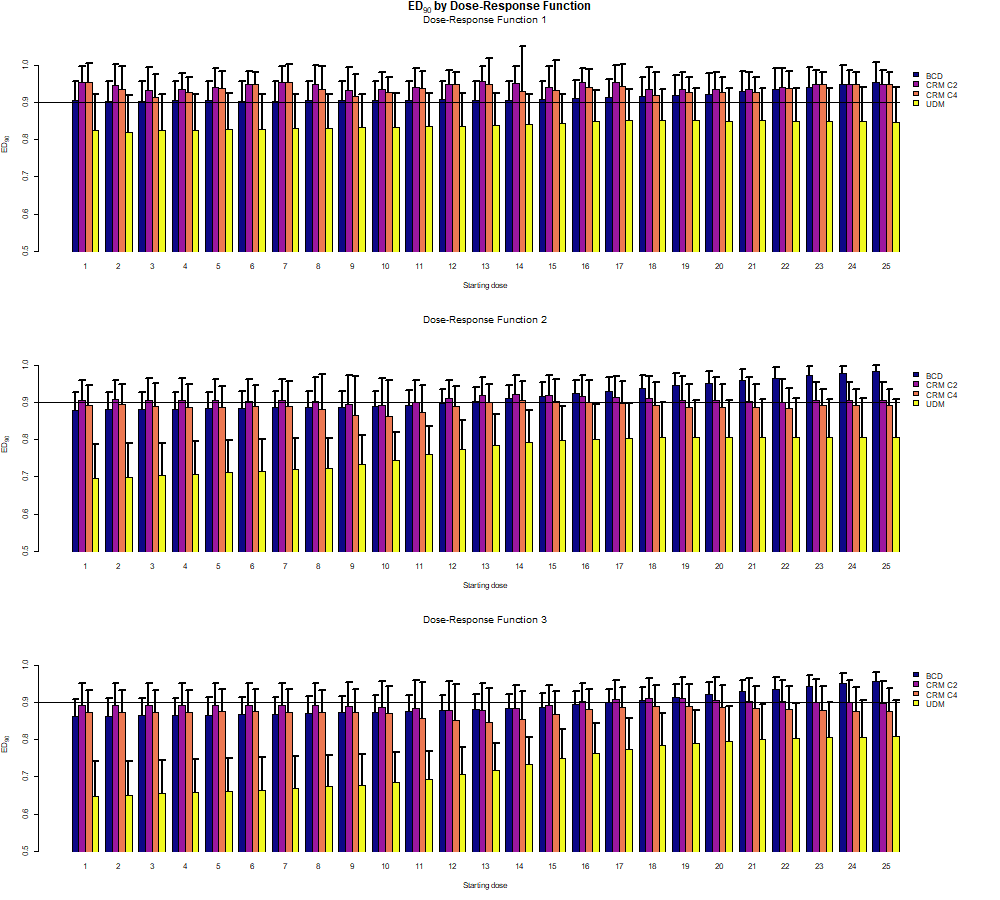


The CRM with cohorts of 2 patients is close to the ED_90_ for sigmoid 2 and 3 (in the ED_90_ range ± 1%), and overestimates ED_90_ for sigmoid 1 between 3% and 5%. CRM with cohorts of 4 patients overestimates ED_90_ for Sigmoid 1 between 1% and 5%; underestimates ED_90_ for sigmoid 2 by 1%-2% and by 2%-4% for sigmoid 3. BCD’s ED_90_’s estimates overestimate ED_90_ when starting doses are above the ED_90_ for the three sigmoid. For the remaining starting doses, the BCD’s estimates of ED_90_ are close to ED_90_ for sigmoid 1. For sigmoid 2 and 3, the estimated ED_90_ are underestimated by 2%-3%, and by 1%-4%, respectively.

**Figure 23. Mean ± Standard Deviation number of observations by dose-response function and by design for ED_90_**


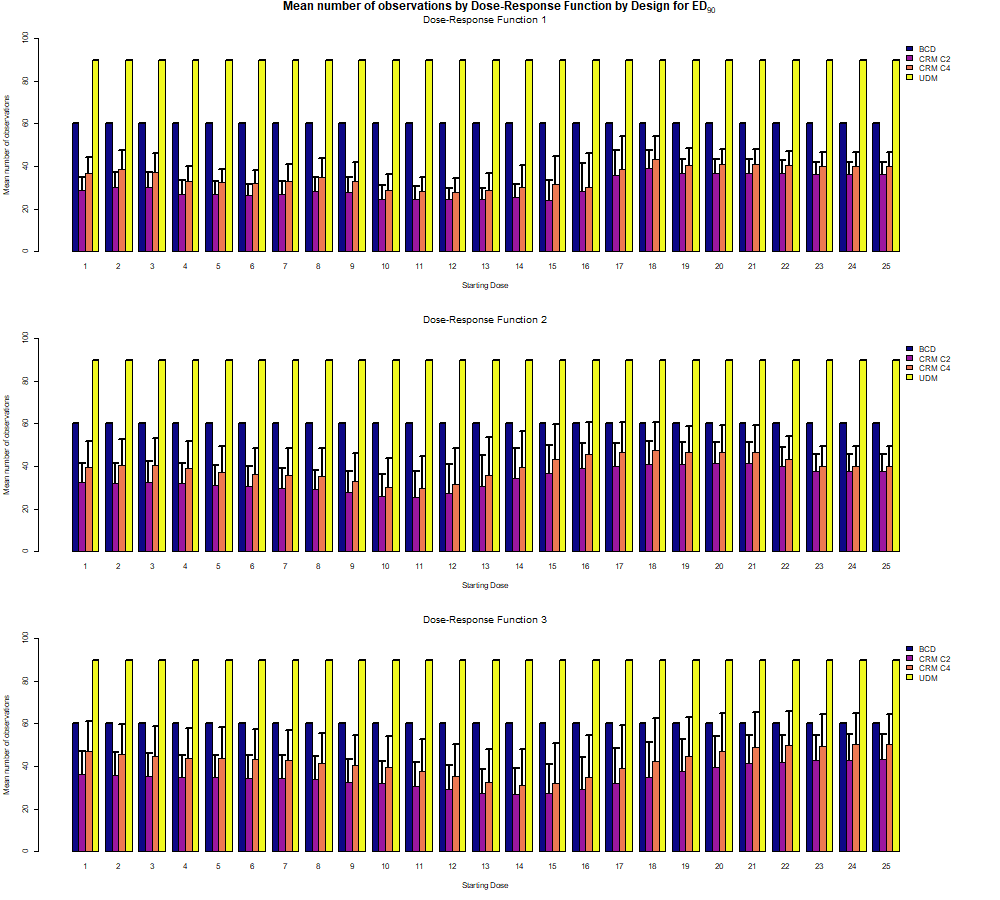


As indicated by the above figure, the BCD needs 60 patients to estimate the ED_90_, where UDM recruits 90 patients to estimate it. For the 3 sigmoid, CRM requires between 20 and 50 observations in average to estimate ED_90_. CRM with cohorts of 2 patients requires between 66% and 33% less observations than CRM with cohorts of 4 patients.

**Figure 24. Mean ± Standard Deviation number of failures dose-response function and by design for ED_90_**


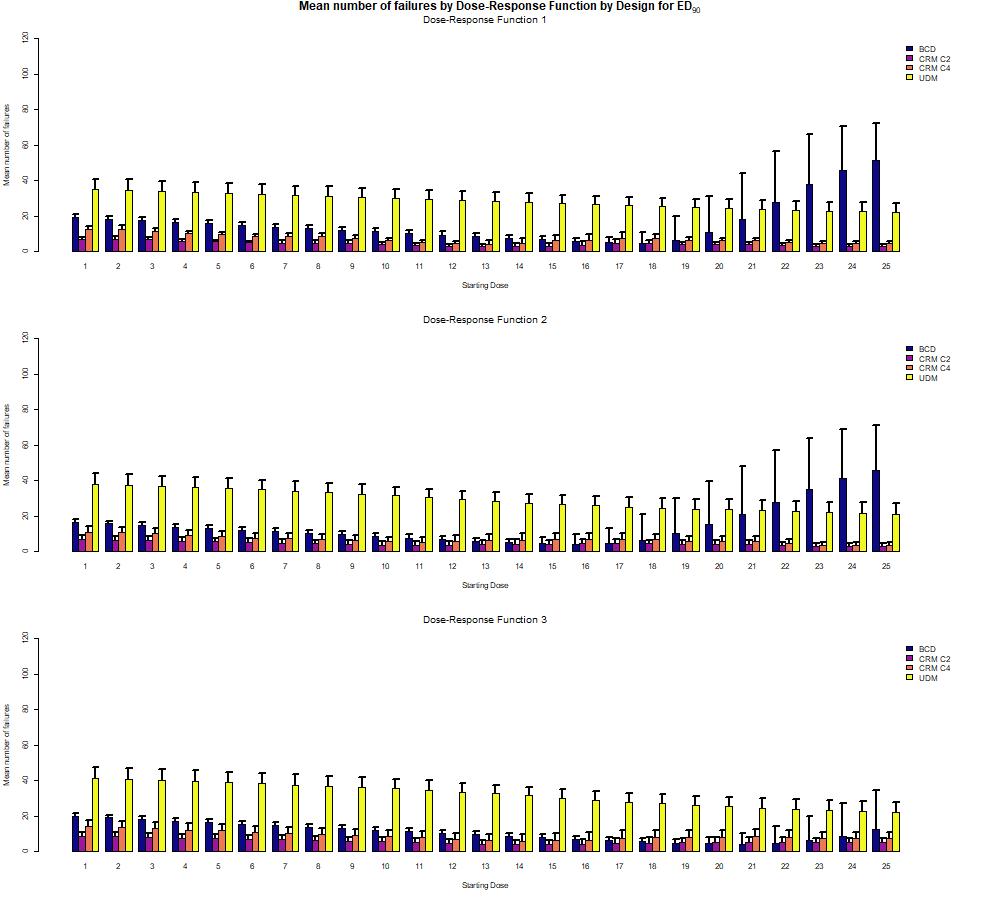


The above figure presents the number of failures when an estimator for the ED_90_ is found. It indicates that the CRM has the lowest mean number of failures on the three sigmoid, compared to UDM and BCD. UDM has a higher number of failures (more than 30) at lowest starting doses, and this number decreases as the starting dose increases. The number of failures for BCD has a ‘U-shape’: it starts with around 20 failures for starting dose 1, then a decrease is observed and the number of failures increases again at higher starting doses. For the three sigmoid, CRM with cohorts of 2 and 4 patients have 6 and 13 failures and then this number decreases as the starting dose increases.

**Bibliography**

1. Dixon W. The Up-and-Down method for small samples. *J Am Stat Assoc*. 1965;60(312):967-978.

2. Dixon W, Massey F. *Introduction to Statistical Analysis. 4th Ed.* McGraw-Hill, New York; 1983.

3. Durham S, Flournoy N. Random walks for quantile estimation. In: *Statistical Decision Theory and Related Topics*. S. Gupta and J. 0. Berger. Springer-Verlag, New York; 1994:467-476.

4. Stylianou M, Flournoy N. Dose finding using the biased coin up-and-down design and isotonic regression. *Biometrics*. 2002;58(1):171-177.

5. Oron AP, Souter MJ, Flournoy N. Understanding Research Methods: Up-and-down Designs for Dose-finding. *Anesthesiology*. 2022;137(2):137-150.

6. Garrett-Mayer E. The continual reassessment method for dose-finding studies: A tutorial. *Clin Trials*. 2006;3(1):57-71.

7. Resche-Rigon M, Zohar S, Chevret S. Adaptive designs for dose-finding in non-cancer phase II trials: influence of early unexpected outcomes. *Clin Trials*. 2008;5(6):595-606.

8. Zohar S, Chevret S. The continual reassessment method: Comparison of Bayesian stopping rules for dose-ranging studies. *Stat Med*. 2001;20(19):2827-2843.

9. Thall PF, Russel KR. A strategy for dose-finding and safety monitoring based on efficacy and adverse outcomes in phase I/II clinical trials. *Biometrics*. 1998;54(1):251-264.

10. O’Quigley J, Pepe M, Fisher L. Continual reassessment method: A practical design for phase I clinical trials in cancer. *Biometrics*. 1990;46(1):33-48.

11. O’Quigley JP. Continual reassessment designs with early termination. *Biostatistics*. 2002;3(1):87-99.

12. Heyd JM, Carlin BP. Adaptive design improvements in the continual reassessment method for phase I studies. *Stat Med*. 1999;18(11):1307-1321.

13. Devlin SM, Iasonos A, O’Quigley JP. Stopping rules for phase I clinical trials with dose expansion cohorts. *Stat Methods Med Res*. 2022;31(2):334-347.
