# Supplementary material for: A Monte Carlo simulation study comparing the up and down, biased-coin up and down and continual reassessment methods used to estimate an effective dose (ED95 or ED90) in anaesthesiology research
Source: BJA Open. 2023 Sep 27;8:100225. doi: 10.1016/j.bjao.2023.100225 (PMC10542596; doi:10.1016/j.bjao.2023.100225)
Supplement: Multimedia component 2 [file mmc2.zip › Appendix1.docx]

**Appendix 1. Description of the R code used for the Monte Carlo simulations**

**R source codes are available for the UDM Monte Carlo simulations:**

1) UDMSimulation.R: random walk for the UDM design;

2) UDMMonteCarlo.R: file generating Monte Carlo simulations for the UDM design for the different starting doses.

**R source codes are available for the BCD Monte Carlo simulations:**

1) BCDSimulation.R: random walk for the BCD design;

2) BCDMonteCarlo.R: file generating Monte Carlo simulations for the BCD design for the different starting doses.

**R source code for the different CRM methods used, available in the main article**

- 1. CRM_power.R (original CRM function written by Pr Zohar & Chevret)
  2. CRMSimulation_RW18pat.R: Random walk 1 for the stopping rule based on the ‘8 patients’ rule
  3. CRMMonteCarloRW18pat.R: file generating Monte Carlo simulations for the CRM Random walk 1 design for the different starting doses with the stopping rule based on the ‘8+1’ patient’s rule
  4. CRMSimulation_RW28pat.R: Random walk 2 for the stopping rule based on the ‘8 patients’ rule
  5. CRMMonteCarloRW28pat.R: file generating Monte Carlo simulations for the CRM Random walk 2 design for the different starting doses with the stopping rule based on the ‘8+1’ patient’s rule

**R source code for the different CRM methods used, available in the supplementary material**

1. CRM_power.R (original CRM function written by Pr Zohar & Chevret)
2. CRMSimulation_RW1CI9515.R: Random walk 1 for the stopping rule based on the confidence interval
3. CRMMonteCarloRW1CI9515.R: file generating Monte Carlo simulations for the CRM Random walk 1 design for the different starting doses with the stopping rule based on the confidence interval
4. CRMSimulation_RW2CI9515.R: Random walk 2 for the stopping rule based on the confidence interval
5. CRMMonteCarloRW1CI9515.R: file generating Monte Carlo simulations for the CRM Random walk 2 design for the different starting doses with the stopping rule based on the confidence interval
6. CRMSimulation_RW18pat.R: Random walk 1 for the stopping rule based on the ‘8 patients’ rule
7. CRMMonteCarloRW18pat.R: file generating Monte Carlo simulations for the CRM Random walk 1 design for the different starting doses with the stopping rule based on the ‘8+1’ patient’s rule
8. CRMSimulation_RW28pat.R: Random walk 2 for the stopping rule based on the ‘8 patients’ rule
9. CRMMonteCarloRW28pat.R: file generating Monte Carlo simulations for the CRM Random walk 2 design for the different starting doses with the stopping rule based on the ‘8+1’ patient’s rule
